# Cell-to-cell natural transformation in *Bacillus subtilis* facilitates large scale of genomic exchanges and the transfer of long continuous DNA regions

**DOI:** 10.1093/nar/gkad138

**Published:** 2023-03-13

**Authors:** Liping Deng, Chao Wang, Xiaoming Zhang, Wenjie Yang, Hao Tang, Xinru Chen, Shishen Du, Xiangdong Chen

**Affiliations:** State Key Laboratory of Virology, College of Life Sciences, Wuhan University, Wuhan 430072, China; State Key Laboratory of Virology, College of Life Sciences, Wuhan University, Wuhan 430072, China; State Key Laboratory of Virology, College of Life Sciences, Wuhan University, Wuhan 430072, China; State Key Laboratory of Virology, College of Life Sciences, Wuhan University, Wuhan 430072, China; State Key Laboratory of Virology, College of Life Sciences, Wuhan University, Wuhan 430072, China; State Key Laboratory of Virology, College of Life Sciences, Wuhan University, Wuhan 430072, China; State Key Laboratory of Virology, College of Life Sciences, Wuhan University, Wuhan 430072, China; State Key Laboratory of Virology, College of Life Sciences, Wuhan University, Wuhan 430072, China

## Abstract

Natural transformation is one of the major mechanisms of horizontal gene transfer. Although it is usually studied using purified DNA in the laboratory, recent studies showed that many naturally competent bacteria acquired exogenous DNA from neighboring donor cells. Our previous work indicates that cell-to-cell natural transformation (CTCNT) using two different *Bacillus subtilis* strains is a highly efficient process; however, the mechanism is unclear. In this study, we further characterized CTCNT and mapped the transferred DNA in the recombinants using whole genome sequencing. We found that a recombinant strain generated by CTCNT received up to 66 transferred DNA segments; the average length of acquired continuous DNA stretches was approximately 27 kb with a maximum length of 347 kb. Moreover, up to 1.54 Mb genomic DNA (37% of the chromosome) was transferred from the donors into one recipient cell. These results suggest that *B. subtilis* CTCNT facilitates horizontal gene transfer by increasing the transfer of DNA segments and fostering the exchange of large continuous genomic regions. This indicates that the potency of bacterial natural transformation is underestimated using traditional approaches and reveals that DNA donor cells may play an important role in the transformation process in natural environments.

## INTRODUCTION

Horizontal gene transfer (HGT) is widely recognized as the driving force for the evolution of prokaryotes (bacteria and archaea) by fostering the exchange of genetic material between microorganisms in a non-vertical manner (1,2). Once inside the cytoplasm of the recipient cells, the transferred DNA may recombine with the recipient chromosome by homologous or site-specific recombination, or stay as extrachromosomal elements, or be degraded if it lacks a site for recombination or an autonomous replication origin. If the transferred DNA is retained in the recipient cells, the physiology and activity of the recipient cells may be significantly altered by genes encoded in the transferred DNA (2,3). It is well documented that HGT contributes to the genomic diversity of bacteria and promotes the spread of pathogenicity islands and antibiotic resistance genes among bacterial pathogens (4,5). Thus, understanding HGT mechanisms advances comprehension of prokaryotic evolution and is critical for controlling pathogens and antibiotic resistance.

There are three canonical mechanisms of horizontal gene transfer in bacteria: conjugation, transduction (including gene transfer agents, a variation of viral transduction) and transformation (6–9). The ability of transferring DNA from one donor bacterium to another by conjugation or transduction is controlled either by plasmids/transposons or by bacteriophages, respectively. However, in the case of transformation, the recipient cell must develop an ability, called competence, to take up and process the extracellular DNA. Therefore, in natural genetic transformation the HGT process is totally controlled by the bacteria (1,2,7). Natural genetic transformation was discovered in 1928 by Frederick Griffith in the human pathogen *Streptococcus pneumoniae* where the exchange of capsule-encoding genomic islands led to serotype switching (10). Currently, approximately 80 bacterial species have been reported to develop natural competence (11), including many human pathogens such as *Haemophilus influenza*, *Vibrio cholerae*, *Neisseria meningitides* and *Acinetobacter baumannii* (12–15).

Natural transformation is arguably one of the best studied HGT pathways and most studies focus on the recipient cell because the exogenous DNA is readily prepared in the laboratory. Most of the genes required for establishing competence and assembly of the DNA uptake machinery have been characterized by studying this process using purified DNA (7,16,17). However, increasing evidence indicates that this classical methodology does not fully recapitulate the transformation events occurring in the natural environment (18,19). It is well known that competence of most naturally transformable bacteria is transient and involves approximately 20–50 proteins, but the sources of extracellular DNA in natural environments are very complex. It is hard to imagine that bacteria would spend so much genetic resources just for passively acquiring free environmental DNA that may or may not be beneficial. In addition, cell-free DNA is prone to degradation by extracellular nucleases in bacterial habitats, which generates fragments that are too short for homologous recombination (20,21). Therefore, it is conceivable that the natural transformation process in the environment depends on other sources of transforming DNA. Recent studies showed that many naturally competent bacteria do not simply uptake environmental DNA from the surroundings. Instead, they actively acquire newly released DNA from healthy, living bacteria via interbacterial predation (22). For example, some *Streptococcus* species co-regulate competence with the secretion of diffusible bacteriocins which lyse nearby sister cells to release their DNA (23), and in swarm boundaries between genetically distinct *B. subtilis* strains, kin discrimination-mediated killing releases DNA from the vulnerable strain for the dominant one (24). Similarly, *V. cholerae* injects type VI secretion system effectors into its neighboring non-kin cells to release their DNA for uptake (25). *Acinetobacter* species, such as *A. baylyi* and *A. baumannii*, also employ interbacterial predation to promote the transfer of large antibiotic resistance islands (26). The coupling of neighbor predation with competence development in the recipients significantly increases transformation efficiency, and in the case of *V. cholerae* fosters the transfer of large genomic regions up to 168 kb (27). This is in stark contrast to transformation with purified genomic DNA where the transferred DNA fragments are usually below 10 kb (28–31). Thus, transformation with living cells as DNA donors is much more efficient than that using purified DNA and it may be more relevant to transformation events in natural environments.

*Bacillus subtilis* is a sporulating soil bacterium that can be isolated from diverse environments and displays great genomic diversity (32,33). It also serves as a model for the study of natural transformation and many other cellular processes of Gram-positive bacteria. *B. subtilis* acquires extracellular DNA when it enters the competence state or K state, which transiently develops in the late exponential growth phase (34,35). Traditionally, purified chromosomal DNA was provided for natural transformation in *B. subtilis*. However, recent studies reported that living cells can also serve as the source of incoming DNA for this species. Distinct *B. subtilis* swarms form a boundary where kin-discrimination-mediated killing promotes horizontal gene transfer between the strains(24). Furthermore, another study showed that surfactin, a biosurfactant and antimicrobial lipopeptides, promotes cell lysis and DNA release which enhances transformation in mixed *B. subtilis* cultures (36). These results indicate that neighbor predation plays an important role in natural transformation of *B. subtilis*. We found that simply spreading a mixture of two naturally competent *B. subtilis* strains carrying different genetic markers on selective Spizizen minimal medium plates resulted in the appearance of recombinants at a very high frequency. This phenomenon was termed cell-to-cell natural transformation (CTCNT) and uses live donor cells as the source of transforming DNA. This is distinct from the standard two-step DNA-to-cell natural transformation (DTCNT) method, which uses purified donor DNA as the transformation material (37). CTCNT is highly efficient and promotes the co-inheritance of distantly localized genetic markers which rarely occurs in DTCNT (37). Moreover, it is reported that CTCNT occurred at a higher frequency under conditions of survival pressure, such as starvation and exposure to some antibiotics, especially kanamycin. Interestingly, CTCNT was almost completely resistant to DNase treatment and appeared to require close proximity between the donor and recipient cells (37). Thus, it was suggested to be a coordinated process between the donor and recipient cells, and the donor cells may play an important role in this process (37). In addition, CTCNT was found to be ubiquitous among *B. subtilis* strains, and occurred in some *Bacillus licheniformis* strains. Therefore, the CTCNT phenomenon may be much more widespread than currently known. However, it is unclear why CTCNT is so highly efficient and how it is regulated currently.

In this study, further characterization of CTCNT in *B. subtilis* revealed that co-inheritance of distantly localized genetic markers was resulted from the transfer and recombination of large continuous DNA segments containing the markers or gene congression. Whole genome sequencing showed that the number of DNA uptake events was much higher in recombinants generated by CTCNT-C (for chromosomal DNA transfer) than those using DTCNT-C. Moreover, the transferred contiguous DNA stretches generated by CTCNT-C were much longer, with a maximum length of 347 kb. In addition, up to 37% of one recipient genome were replaced by the homologous sequence from the donor in a single CTCNT-C experiment, indicating that CTCNT-C promotes large scale genetic exchanges. These results suggest that CTCNT-C facilitates horizontal gene transfer in *B. subtilis* by increasing the number of transfer events and fostering the transfer of large continuous genomic regions. This powerful HGT strategy may contribute to the genomic diversity of *B. subtilis* and its adaption to various environments.

## MATERIALS AND METHODS

### Bacterial strains, plasmids and growth conditions

The bacterial strains and plasmids used in this study were described in Supplementary Table S1. Bacteria were cultured at 37°C for all experiments. *Escherichia coli* was grown in lysogeny broth (LB) or on LB agar plates (1.5% agar), *B. subtilis* was cultured in LB or minimal media (MM) (38) (on agar plates with 1.5% agar). Amino acids were added to MM at the following concentration when needed: tryptophan (Trp: 20 μg/ml), lysine (Lys: 50 μg/ml), phenylalanine (Phe: 50 μg/ml), histidine (His: 50 μg/ml), methionine (Met: 50 μg/ml), cysteine (Cys: 50 μg/ml). The medium was supplemented with kanamycin (30 μg/ml for LB, 50 μg/ml for MM), ampicillin (100 μg/ml), erythromycin (2 μg/ml), and chloramphenicol (7.5 μg/ml for LB, 50 μg/ml for MM) if required.

### Construction of plasmids

All plasmids used for the generation of gene deletions and replacements were derivatives of the *E. coli–B. subtilis* shuttle vector pNNB194. (i) Plasmids for in-frame markerless deletion of genes. First, two homologous arms of approximately 350 bp were amplified by PCR from the genomic DNA of *B. subtilis* 168 using primer pairs *gene*-US/*gene*-UA and *gene*-DS/*gene*-DA for any given gene, respectively (Supplementary Table S2). These two homologous arms were joined together by overlap extension PCR (SOE-PCR) using primer pairs *gene*-US and *gene*-DA. The DNA fragment was subcloned into vector pNNB194 by digestion and ligation. The resulting plasmid was verified by sequencing and designated as pNNB194-Δ*gene*. (ii) Plasmid pNNB194-*trp* was constructed for replacement of the *trpC2* allele in *B. subtilis* 168. The open reading frame of *trpC* was amplified by PCR from the genomic DNA of *B. subtilis* BG2036 by primer pairs *trpAF*-S and *trpAF*-A. The DNA fragment was subcloned into vector pNNB194 by digestion and ligation and the resulting plasmid was verified by sequencing. (iii) Plasmids for integrating *mCherry* into the chromosome. First, two homologous arms of approximately 350 bp were amplified by PCR from the genomic DNA of *B. subtilis* 168 using the primer pairs indicated in Supplementary Table S2. The coding sequence of P*_pen_-mCherry* and P*_pen_-gfp-mut2* was amplified by PCR with primers. The three DNA fragments were joined by SOE-PCR and subcloned into pNNB194 by digestion and ligation. The resulting plasmids were verified by sequencing and designated as pNNB194-mc-c, pNNB194-mc-s, pNNB194-mc-y, pNNB194-gfp, respectively. (iv) pGK12H. The multiple cloning site of plasmid pUC18 was amplified by PCR by the primer pairs MCS-S/MCS-A, and the PCR products were digested with HpaII and ligated into HpaII-digested pGK12.

### Construction of strains

The donor strains CAmC, SAmC, YAmC and the recipient strains were derived from *B. subtilis* 168. Genes were knocked out/in as previously described (39). Briefly, pNNB194 derivatives harboring the homologous arms for the genes to be knocked out were electrotransformed into competent *B. subtilis* cells, and transformants were selected by erythromycin resistance (2 μg/ml). Transformants were verified by PCR using the primer pairs pNNB194-seqS/pNNB194-seqA (Supplementary Table S2) and cultured in LB medium containing erythromycin (2 μg/ml) at 45°C for 8 h. pNNB194 derivatives cannot replicate at 45°C due to the temperature-sensitive replicon; therefore, they integrate into the chromosome at the locus of the gene to be knocked out. One hundred μl of the integrant grown at 45°C was plated on LB agar plates containing erythromycin (2 μg/ml), and further verified by PCR with the primer pairs *gene*-seq S/*gene*-seq A. The correct colonies were picked up and cultured in LB medium at 37°C for 12 h, then diluted 1:100 in fresh LB medium and grown at 37°C for 12 h. This culturing procedure was repeated seven times to promote the double crossover. After serial transfer without antibiotics, cells were plated on LB agar plates, and checked for kanamycin sensitivity. Kanamycin sensitive colonies were confirmed using the primer pairs *gene*-seq S/*gene*-seq A to ensure that the gene was deleted. (i) Construction of strains carrying P*_pen_-mCherry*. *B. subtilis* 168 was firstly cured of the natural tryptophan deficiency with pNNB194-*trp*. *B. subtilis* 168 *trp^+^*was then transformed with pNNB194-Δ*comK* to create *B. subtilis* 168 *trp^+^* Δ*comK*, which was transformed with plasmid pNNB194-mc-c, pNNB194-mc-s and pNNB194-mc-y to construct CAmC, SAmC and YAmC strains, respectively. (ii) Construction of *B. subtilis* TLPHMC and its derivatives. *lysA*, *hisD*, *pheA*, *metC* and *cysE* were consecutively deleted in *B. subtilis* 168 using pNNB194 derivatives. Deletion of each gene was carried out as described above. The recipient was distinguished from the donor harboring *mCherry* by inserting P*_pen_-lacIΔ11-gfp-mut2* into the *amyE* locus of TLPHMC (TLPHMC-G) by double crossover (using pNNB194-gfp). Plasmid pGK12H was transformed into TLPHMC(-G) for the study of plasmid transfer by CTCNT and DTCNT.

### Preparation of chromosomal and plasmid DNA

Chromosomal DNA was purified from the pellet of 5 ml *B. subtilis* culture grown overnight in LB. Genomic DNA was extracted using the TIANamp bacteria DNA kit (Tiangen) according to the manufacturer's protocol and extracted genomic DNA was stored at −20°C prior to use. The same experimental procedure was used for plasmid extraction with TIANprep mini plasmid kit (Tiangen).

### Natural transformation assay

Cell-to-cell gene transfer in *B. subtilis* allows both chromosomal and plasmid DNA transfer. All the cell-to-cell natural transformation with chromosome (CTCNT-C) assays were carried out using the direct plating method as previously described (37). Briefly, donor (prototroph, kanamycin sensitive) and recipient (auxotrophic, kanamycin resistant) strains were inoculated separately into LB and LB supplemented with kanamycin (30 μg/ml), cultured with shaking at 37°C overnight. The donor cell culture was diluted 1:100 (vol/vol) into fresh liquid LB and incubated with shaking at 37°C for 6 h, when the cell density was up to 1 × 10^8^ cells/ml. At this cell density, the DNA concentration of the culture was estimated to be about 0.46 μg/ml (40). At the same time, an overnight culture of the recipient strain was similarly diluted into fresh liquid MM supplemented with appropriate amino acids and kanamycin and incubated with shaking at 37°C for 11 h. Note that by culturing the donor strain in LB medium, which does not support competence development, and culturing the recipient in MM medium, which supports competence development, we could ensure the recombinants were almost all derived from the recipient strain. Equal volume of donor and recipient cultures were mixed and 100 μl of the mixture was inoculated onto MM plates containing appropriate amino acids and kanamycin (50 μg/ml). The natural transformation assay using purified chromosomal DNA (with different DNA concentrations of 0.5, 5 or 10 μg/ml) was performed by mixing an equal volume of donor chromosomal DNA solution with the recipient cell culture (DNA-to-cell natural transformation with purified chromosome, DTCNT-C). 100 μl of the mixture was plated onto selective plates and incubated at 37°C for 36 h to select for recombinant strains. The cell-to-cell natural transformation with plasmid (CTCNT-P) or the DNA-to-cell natural transformation with purified plasmid (DTCNT-P) was performed using the same method as CTCNT-C or DTCNT-C. Strain 168 was used as the recipient, whereas TLPHMC-G harboring pGK12H was provided as the donor strain. Transformants were selected on MM plates supplemented with chloramphenicol and tryptophan. Note that CTCNT and DTCNT assays in this study are different than the standard two-step transformation assay which requires treatments of the recipient cells to induce the competence state.

### Quantitation of transformation efficiency

**Figure 1.**
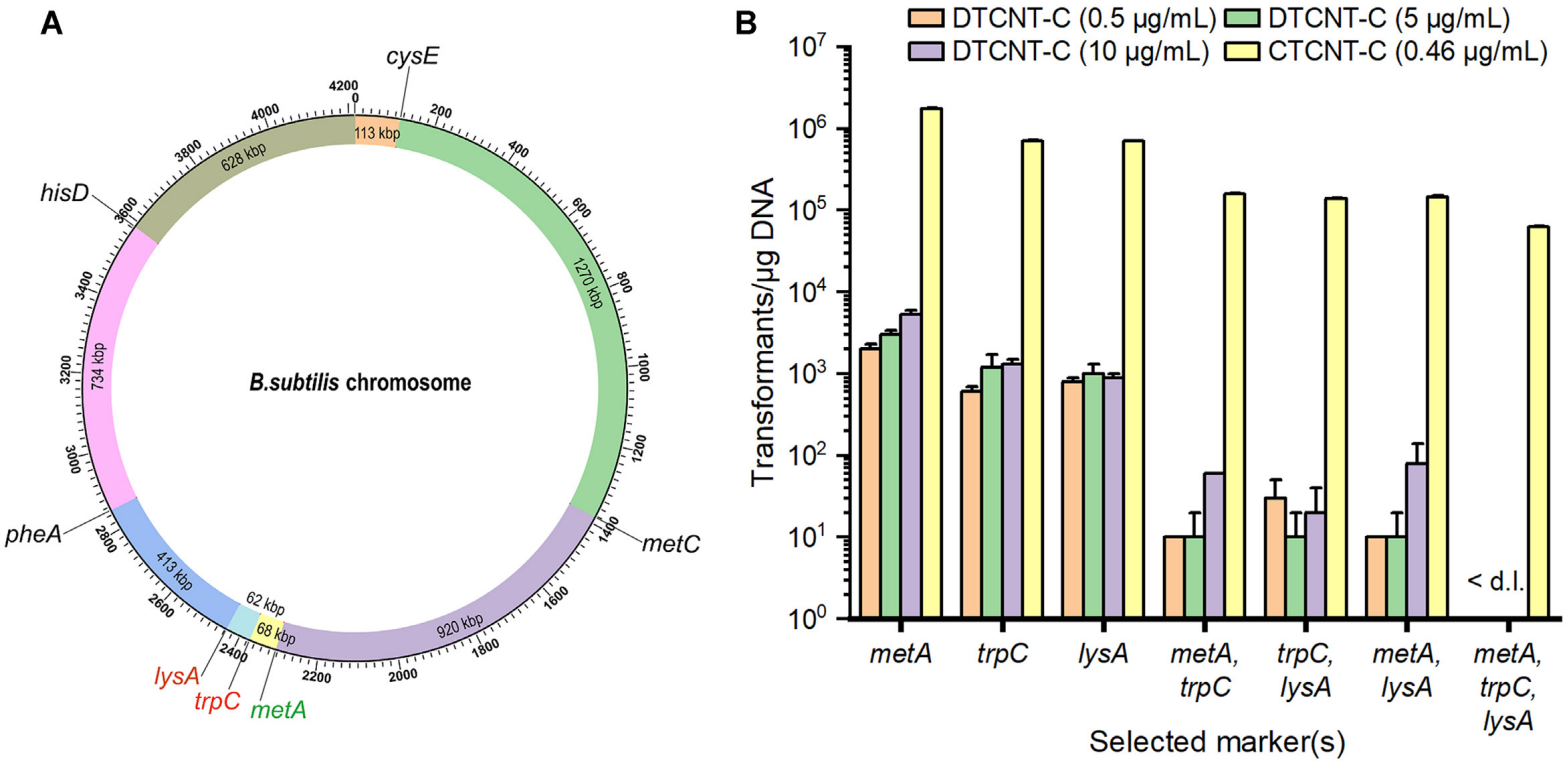
Efficiency of natural transformation in *B. subtilis* using live donor cells or purified chromosomal DNA. (**A**) Genetic positions of the auxotrophic markers in the *B. subtilis* chromosome. The whole chromosome of *B. subtilis* is approximately 4,200 kb and the genetic distance between the auxotrophic genes *cysE*, *metC*, *metA, trpC*, *lysA*, *pheA* or *hisD* are indicated. *trpC*, *lysA* and *metA* are mutated in BR151/pBE2, whereas *cysE*, *metC*, *trpC*, *lysA*, *pheA* or *hisD* are mutated in TLPHMC(-G)/pBE2. (**B**) Comparison of the efficiency of DTCNT-C and CTCNT-C. Live BG2036Δ*comK* (prototroph) cells or purified BG2036Δ*comK* chromosomal DNA was used as the source of transforming DNA, and BR151/pBE2 (auxotrophic for Trp, Lys, and Met) was used as the recipient. Exponentially growing BG2036Δ*comK* was mixed with an equal volume of BR151/pBE2 growing in MM, and 100 μl of the mixture was plated on MM with kanamycin in the absence of one-, two-, or three of the amino acids required for BR151/pBE2 growth. The total DNA concentration of live donor cells in CTCNT-C experiments was estimated to be 0.46 μg/ml. Therefore, purified chromosomal DNA was used at an equivalent concentration, 5 μg/ml or 10 μg/ml in DTCNT-C assays. Recovered recombinant strains and the efficiency of transformation are represented on the X-axis and Y-axis, respectively. Due to the great efficiency of CTCNT, the number of transformants for selection of one marker could not be counted accurately so the values were estimated; the actual values should be higher. < d.l., below detection limit.

**Figure 2.**
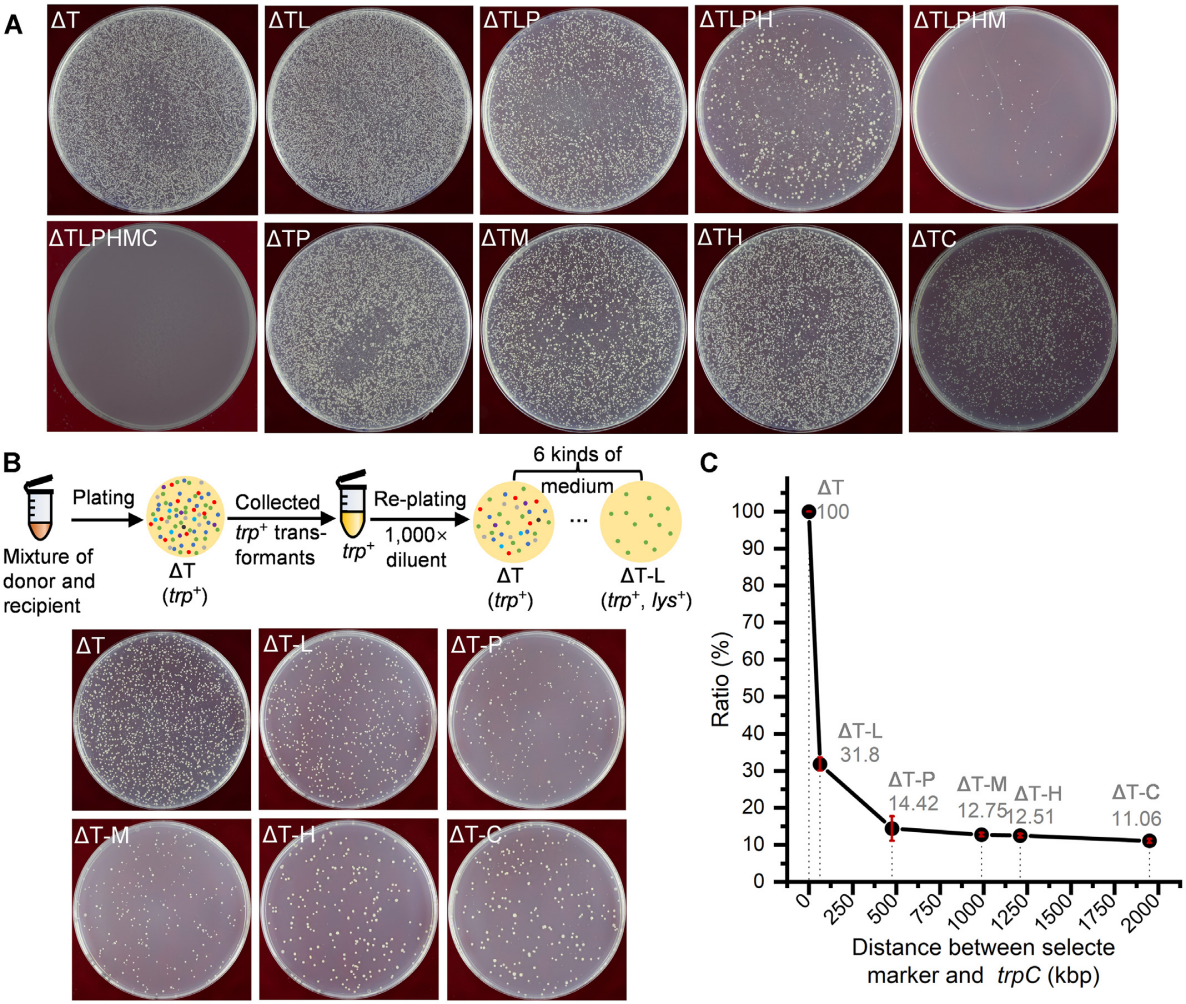
Co-inheritance of multiple genetic markers in *B. subtilis* using CTCNT-C. BG2036Δ*comK* and TLPHMC/pBE2 strains were used as the donor and recipient, respectively. The letter(s) on each plate represent the omitted amino acid(s): T for Trp, L for Lys, P for Phe, H for His, M for Met, and C for Cys. (**A**) Recombinant strains obtained on selective plates lacking the indicated amino acids. (**B**) Unselected marker co-inherited with *trpC*. A schematic diagram for the experiment was shown above the plates. Transformants obtained on MM plates lacking Trp were pooled together, diluted 1,000 fold, and plated on selective plates lacking the indicated amino acids. (**C**) The ratio of co-inheritance of the unselected marker with *trpC*. The co-inheritance ratio decreased as the distance between *trpC* and the unselected marker increased if they were within the range of 500 kb. Above 500 kb, the ratio of co-inheritance was lower and constant irrespective of the distance. The X-axis and Y-axis show the distance from the unselected marker to *trpC* and the ratio of co-inheritance, respectively. The ratio of co-inheritance was calculated using data from Figure 2B and Supplementary Figure S1.

**Figure 3.**
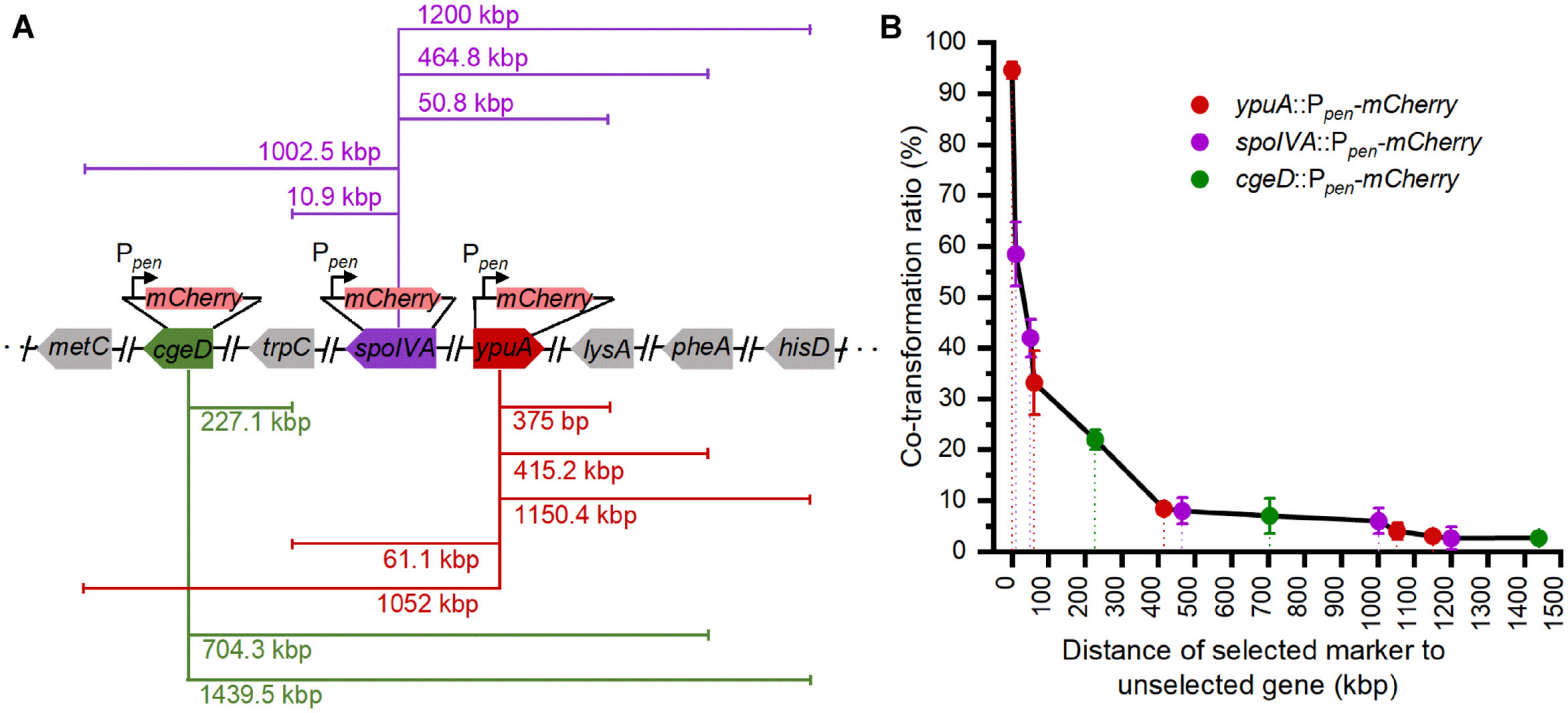
Fluorescent protein genes as the unselected markers co-transferred with selected markers. (**A**) Schematic diagrams of *mCherry* (pink pentagons) inserted into the donor chromosome at different locations and the distance from *mCherry* to selected auxotrophic genes (grey pentagons). All strains were *B. subtilis* 168 derivatives. CAmC, SAmC and YAmC indicated that *mCherry* was inserted into *cgeD* (green pentagon), *spoIVA* (purple pentagon), and *ypuA* (red pentagon), respectively. The distance between *mCherry* and 5 auxotrophic markers was indicated. (**B**) The ratio of co-inheritance of *mCherry* with the selected markers decreased as the distance between *mCherry* and the markers increased if they were within the range of 500 kb. Above 500 kb, the ratio of co-inheritance was lower and constant. The Y-axis indicates the co-transferred ratio, while the X-axis indicates the distance between *mCherry* and the selected auxotrophic markers. The color of the circles is consistent with that in (A). Data represent the average of three independent biological experiments (±SD, as depicted by the error bars).

Different approaches were used to calculate the transformation efficiency, depending on the design of experiments. In Figure 1B, the number of recombinants on the selective plates (selection for one or two genetic markers) were too many to be counted accurately because CTCNT-C was highly efficient. As a result, we estimated the number of transformants by counting the number of transformants in a unit area. The concentration of DNA of the donor cell culture was estimated to be about 0.46 μg/ml, (about 1 × 10^8^ cells/ml) (40). Based on this estimation, purified chromosomal DNA was added to the recipient cells at a final concentration of 0.5, 5 or 10 μg/ml for DTCNT-C assay. We used transformants/μg DNA as the unit to compare the transformation efficiency of CTCNT and DTCNT. In Figure 2C, the unselected markers were amino acid prototroph, we determined the ratio of co-inheritance accurately by plating the *trp^+^* transformants on two kinds of MM plates for selection of *trp^+^* recombinants or recombinants of *trp^+^* with another marker. The ratio of co-inheritance was calculated by the No. of double recombinants versus the No. of *trp^+^* recombinants. In Figure 3B, we could not directly select for the unselected marker *P_pen_-mCherry*. Instead, we screened the transformants for the presence of *P_pen_-mCherry* by PCR when a single marker was selected on MM plates (∼480 transformants per group). The transformation efficiency was calculated as the number of transformants with *P_pen_-mCherry* versus the number of tested transformants.

### Fluorescence microscopy and analysis

The donor SAmC strain and the recipient TLPHMC-G/pBE2 cells were prepared following the CTCNT-C procedure described above. A 100 μl aliquot of the mixture was plated onto MM plates lacking lysine (or tryptophan; methionine; histidine; cysteine) and kanamycin, and cultured at 37°C for 12 h. The colonies were collected using 1 mL PBS and the suspension was concentrated by centrifugation. The cells were immobilized on a glass slide for photographing. All phase contrast- and fluorescence microscopy images were acquired using an Olympus BX53 upright microscope with a Retiga R1 camera from QImaging, a CoolLED pE-4000 light source, and a U Plan XApochromat phase contrast objective lens (100×, 1.45 numerical aperture [NA], oil immersion). Green- and mCherry fluorescence signals were visualized using the Chroma EGFP filter set (EGFP/49002) and mCherry/Texas Red filter set (mCherry/49008), respectively with an exposure time of 0.6 s. Images were analyzed for the number of donors, transformants and recipients using ImageJ software.

### Genome sequencing and assembly

The genomes of RO-NN-1 and TLPHMC/pBE2 were sequenced using the PacBio Sequel II Platform (20 kb library) and short read paired-end sequencing (150 bp in length) with an Illumina HiSeq instrument at an average depth of 300. The obtained reads were used to assemble the genome with Canu (41) and the Illumina sequencing data were further used to improve the assembly. The RO-NN-1 circular chromosome is 4,011,967 bp, while TLPHMC/pBE2 contains a 4,215,071 bp circular chromosome and a 6,299 bp circular plasmid.

### Whole-genome sequencing of transformants

Genomes were sequenced by Illumina HiSeq next-generation sequencing (NGS). The 33 transformants recovered from natural transformation assays described above were grown at 37°C overnight in LB medium. Genomic DNA was isolated from a 5 ml aliquot using the HiPure bacterial DNA kit (Magen) according to the manufacturer's instructions. The quality of isolated DNA samples was verified, and non-degraded samples were used to prepare the DNA libraries. Paired-end sequencing was performed on an Illumina HiSeq 2500 with an average depth of 300.

### Genome comparison

The genome was segmented into contiguous fragments of 10 kb and aligned to the corresponding reference using blastn (2.9.0+). The best alignment was retained and extended for 5 kb upstream and downstream. Each fragment was globally aligned to the extended aligned fragment using EMBOSS needle (6.6.0.0) with params ‘-gapopen 10 -gapextend 0.5 -endweight -endopen 0 -endextend 0’. The number of differences per 10 kb was evaluated by counting the number of events necessary to mutate the reference to obtain the 10 kb fragment. The outputs were visualized using the R package circlize (0.4.11); The scripts used for genome comparison are available on FigShare at https://doi.org/10.6084/m9.figshare.22081127.v1.

### Scoring of horizontal gene transfer events

Illumina Hiseq raw data files were analyzed by CASAVA (base calling), converted into raw sequences (raw reads), and stored in FASTQ file format. Raw reads from each library were evaluated using FastQC (version 0.11.8) with the linker sequence and low-quality bases trimmed using Trimmomatic (version 0.39). Filtered paired reads of recombinant genomes were mapped against reference genomes using BWA (version 0.7.17) (option mem). The reference genomes of *B. subtilis* RO-NN-1 and TLPHMC were newly sequenced. SAMtools (version 1.9) was used to compare the results from BWA output files for format conversion and sorting. MarkDuplicates of GATK (version 4.1.1.0) was used to mark repeated sequences. Statistical analysis of deep coverage was performed using BEDTools (version 2.28.0). GATK’s HaplotypeCaller was used to analyze genotype differences between each recombinant genome and reference genome. Poor quality- or insufficiently covered single nucleotide polymorphisms (SNPs) were filtered out and only the homozygous variants were retained. The transformation events were defined as contiguous runs of positions with donor-specific variants. The transfer segments were sequentially inferred in the direction of increasing genomic coordinates. The start position of such a continuous stretch was initially recorded with two consecutive donor variation sequences, and continuous donor-variation clusters were searched by extending from this seed position as far as possible. The end coordinate was determined by the last continual donor nucleotide interrupted by the recipient-specific sequence. The start and end positions of such continuous stretches were kept for output if the length exceeded 500 nucleotides. The output was visualized using TBtools (42).

### Determination of plasmid copy number

*B. subtilis* strain TLPHMC-G/pGK12H was cultured overnight in LB, cells were collected by centrifugation and resuspended in 20 mg/ml lysozyme solution (Biosharp), subsequently placed in the thermostat water bath for 30 min at 37°C, then as the DNA template for real-time fluorescent quantitative PCR (qPCR) analysis to determine the relative plasmid copy number to chromosome. Primer pairs GK12H-S/GK12H-A and *rpoB*-S/*rpoB*-A *rpoB*-S/*rpoB*-A were used to represent pGK12H plasmids and an internal reference of chromosome, respectively. The qPCR data were analyzed by the 2^–Δ^*^CT^* method (43). Three independent experiments were performed.

## RESULTS

### *B. subtilis* CTCNT promotes the co-inheritance of distantly localized markers via transfer of large continuous DNA fragments or co-transfer of discrete DNA fragments

In previous studies, we observed that three genetic markers, *metA*, *trpC* and *lysA* residing in a DNA segment of 130 kb (Figure 1A, the distance between *metA* and *lysA*), could be simultaneously transferred into the recipient cells through a single CTCNT-C experiment (37), indicating that CTCNT-C promotes the transfer of large chromosomal DNA segments. However, how this was achieved was not known. To further characterize this phenomenon, we quantified the transformation efficiency upon selection for one or multiple of these genetic markers simultaneously by the CTCNT-C assay. When one marker was selected, the transformation efficiencies using live cells as DNA donors were at least two orders of magnitude higher than those using saturating concentrations of purified DNA (Figure 1B). Surprisingly, the CTCNT-C transformation efficiency was only slightly reduced when two or three markers were selected simultaneously (*metA* and *trpC*, *trpC* and *lysA, metA* and *lysA*). By contrast, transformation frequency was drastically reduced using purified DNA for transformation assays if two or three markers were simultaneously selected, even using a 10-fold higher free DNA concentration compared with that of the donor cell (10 μg/ml versus 0.46 μg/ml).

To test if genetic markers separated by a longer distance (>200 kb) could be co-inherited in CTCNT-C, a mutant *B. subtilis* strain auxotrophic for six amino acids due to spontaneous mutations in *trpC* and in-frame markerless deletions of *lysA*, *pheA*, *hisD, metC* and *cysE* was constructed (TLPHMC). The distances between the mutated genes ranged from 62 kb up to almost 2,000 kb (Figure 1A). Similar to the results above, many transformants were obtained by CTCNT-C upon selection for two markers regardless of the distance between the markers (Figure 2A and Supplementary Figure S1A; compare the number of colonies on the plates lacking TL with those lacking TP, TM, TH and TC). Interestingly, transformants were also obtained on selective plates lacking 3–5 of the 6 amino acids required for the growth of the recipient strain, although the number of transformants decreased as the selective pressure increased. These results indicate that CTCNT-C is very effective in horizontal gene transfer and promotes the co-inheritance of multiple distantly localized genetic markers.

Since multiple markers were simultaneously selected in the above experiments, the efficiency of co-inheritance might be skewed. Therefore, we tested if unselected markers would co-inherited with the *trpC* selection marker by CTCNT-C. Transformants on plates lacking tryptophan (*trp^+^*) were pooled together, diluted 1,000 fold, and re-plated on MM plates lacking Trp and one or more of the indicated amino acids, respectively. As shown in Figure 2B and Supplementary Figure S2, many *trp^+^* recombinants were able to form colonies on MM plates lacking 2–5 of the 6 amino acids required for the growth of the recipient strain, indicating that the unselected markers were co-inherited with *trpC*. Consistent with the results of direct selection in Figure 2A, the number of colonies on the plates decreased as the selective pressure increased (Supplementary Figure S2).

The ratio of co-inheritance of an unselected marker with *trpC* was calculated by the number of double recombinants versus the number of *trp^+^* recombinants. As shown in Figure 2C, the ratios of co-inheritance of *trpC* with *lysA* (62 kb), *pheA* (475 kb), *metC* (988 kb), *hisD* (1,209 kb) and *cysE* (1,950 kb) were 31.8%, 14.42%, 12.75%, 12.51% and 11.06%, respectively (Figure 2C). Plotting the ratios of co-inheritance versus distances between the unselected markers with *trpC* showed that the ratios of co-inheritance of the unselected markers with *trpC* decreased sharply as their distances to *trpC* increased in the range of 0–500 kb (distance between *trpC* and *pheA*), but stayed constant around 10–15% if the distance was above 500 kb. The co-inheritance of *trpC* and *cysE*, which were separated by almost half of the chromosome, was likely due to two independent recombination events (double transformation). If the ratio of co-inheritance of *trpC* and *cysE* (11.06%) was considered as the double transformation ratio, and the ratio of inheritance of selected *trpC* was 100%, the mechanism of co-inheritance for any unselected marker with *trpC* could be roughly determined. The co-inheritance ratio of *trpC* and *lysA* (31.8%) suggested that a significant fraction of the *trpC^+^ lysA^+^* recombinants was likely due to the recombination of continuous DNA segments harboring *trpC* and *lysA* into the chromosome, although double transformation was probably the dominant mechanism. On the other hand, the co-inheritance of *trpC* with *pheA*, *metC* or *hisD* was likely largely due to two recombination events with rare cases resulted from the recombination of a large consecutive DNA segment carrying both markers, as their co-inheritance ratios were only slightly higher than the double transformation ratio 11.06%. Based on this analysis we could deduce that genetic markers within a distance of 62 kb could be transformed simultaneously through a continuous DNA fragment in CTCNT-C, whereas those separated by >475 kb are rarely transformed integrally through single recombination event.

### Co-inheritance of an unselected marker with a selected marker within the range of 500 kb decreases drastically as the distance between them increases

To further study the relationship between the co-inheritance ratio and the distance between the selected and unselected markers, the co-inheritance of auxotrophic markers with an exogenous unselected marker integrated into the *B. subtilis* chromosome were examined using CTCNT-C. *mCherry* encoding red fluorescent protein was integrated at three different locations: *cgeD*, *spoIVA* and *ypuA*, under the constitutive promoter P*_pen_* and the resulting strains were named CAmC, SAmC and YAmC, respectively. The distance of *mCherry* to the auxotrophic markers ranged from a few hundred bp to 1,439 kb. The recipient strain TLPHMC/pBE2 was labeled with GFP by integrating *lacI-gfp* under the P*_pen_* promoter at the *amyE* locus. The co-inheritance of P*_pen_-mCherry* with the auxotrophic markers was examined by PCR (480 transformants were examined in each group). Up to 94.61% of *lys^+^* transformants carrying *ypuA*::P*_pen_-mCherry* (Figure 3 and Supplementary Figure S3), which was expected since *ypuA* was only 375 bp away from the *lysA* gene. However, the co-inheritance ratio decreased drastically as the distance between the auxotrophic marker and *ypuA*::P*_pen_-mCherry* increased. Approximately 33.19% of the *trp^+^* transformants carried *ypuA*::P*_pen_-mCherry* (distance between *trpC* and *ypuA*::P*_pen_-mCherry:* 61.1 kb), whereas only ∼8.39% of the *phe^+^* transformants harbored *ypuA*::P*_pen_-mCherry* (distance between *pheA* and *ypuA*::P*_pen_-mCherry*: 415.2 kb) (Figure 3A and B). Co-inheritance of *ypuA*::P*_pen_-mCherry* with the auxotrophic markers was detected at a very low frequency when the distance between them was above 1,000 kb. Similar results were obtained when P*_pen_-mCherry* was integrated at the *spoIVA* locus or the *cgeD* locus. Plotting the ratios of co-inheritance with the distances between *P_pen_-mCherry* with the selected markers showed that the co-inheritance frequency dropped significantly as the distance between the unselected and the selected markers increased within the range of 500 kb, however, when the distance was above 500 kb, the co-inheritance ratio stayed constant at a very low frequency (Figure 3B).

**Figure 4.**
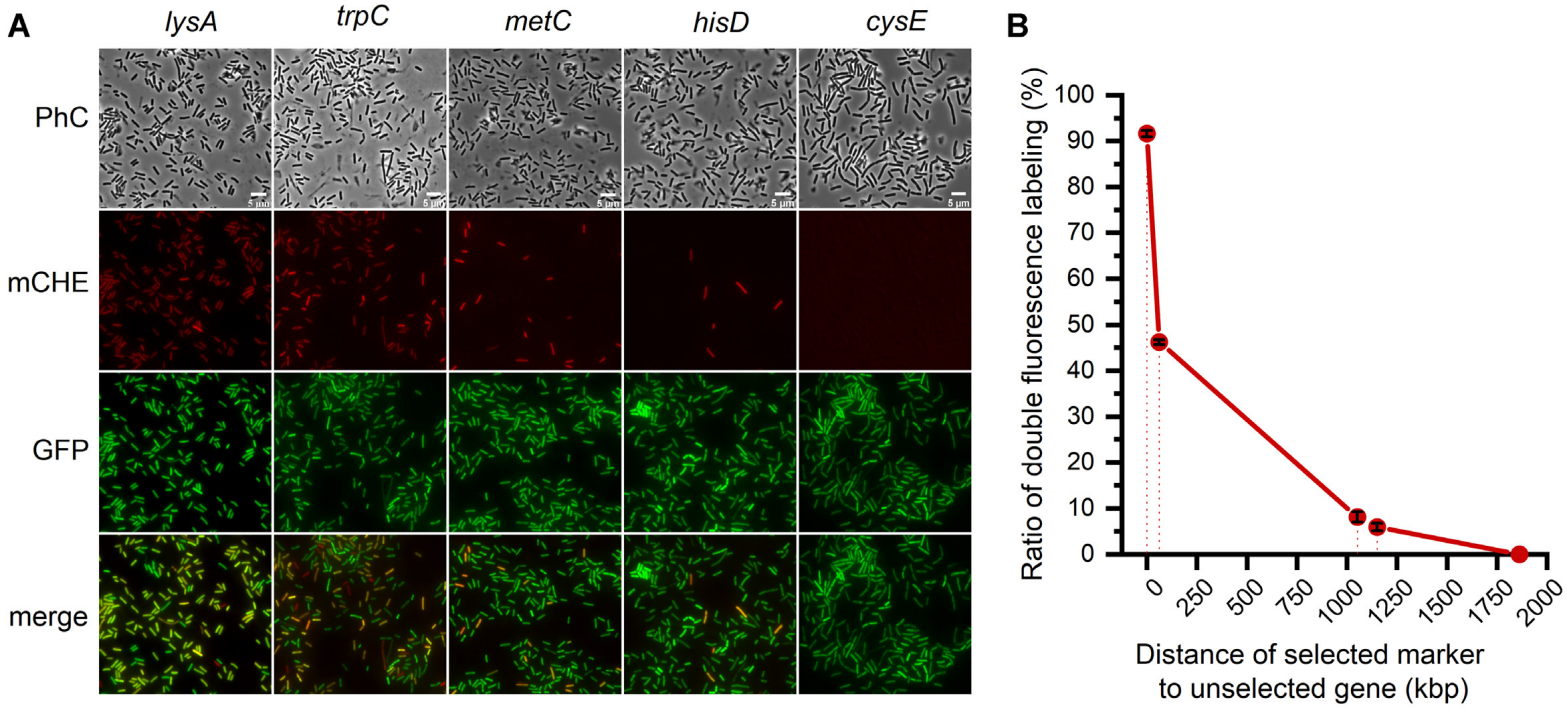
Dual labeling of *B. subtilis* with fluorescent proteins to monitor the co-inheritance of unselected markers with selected markers. CTCNT-C assays were performed using strain YAmC (*mCherry* inserted at *ypuA*) as the donor strain and TLPHMC-G/pBE2 (harboring a *gfp* gene at the *amyE* locus) as the recipient strain. (**A**) Representative fluorescence images of recombinant cells. Markers on top of the images indicate the selected marker. PhC, phase contrast; mCHE, mCherry channel; GFP, GFP channel; merge, merge of mCherry- and GFP channel. Scale bar, 5 μm. (**B**) The ratio of transformants with dual fluorescence. Distance from the *mCherry* gene to the selected auxotrophic markers is indicated on the X-axis.

The co-inheritance of *ypuA*::P*_pen_-mCherry* with the selected markers was also evaluated by the appearance of red fluorescent transformants. Most of the *lys^+^* transformants displayed red fluorescence, whereas only approximately 50%, 10%, or <10% of the *trp^+^*, *met^+^* or *his^+^* transformants expressed mCherry (Figure 4). These results were consistent with the above results that co-inheritance of genetic markers frequently occurs in CTCNT-C, but the efficiency decreased as the distance between *P_pen_-mCherry* and the marker increased in a range of 500 kb and then stayed constant at a low frequency.

### Comparative genomic analysis of recombinants generated by CTCNT-C reveals high transfer frequency of large genomic regions

To determine the underlying mechanism for the high efficiency of CTCNT-C, whole genome sequencing (WGS) was used to determine the lengths of the transferred DNA segments and the number of recombination events in transformants obtained by CTCNT-C. Two distinct *B. subtilis* strains, RO-NN-1 and TLPHMC/pBE2, were used as the donor and recipient strains, respectively. Strain RO-NN-1 was isolated from the Mojave Desert (44) and strain TLPHMC/pBE2 is a derivative of 168. As shown in Figure S4, the two strains shared a 3.73 Mb core genome, but there were unique accessory genomes of 0.23 Mb in RO-NN-1 and 0.48 Mb in TLPHMC/pBE2, respectively. The average nucleotide identity (ANI, based on MUMmer algorithm) of the two genomes was 98.08% and the synteny shows a high degree of conservation. However, the two strains contained significant differences in genomic features and SNPs. RO-NN-1 was distinguished from TLPHMC/pBE2 by 70,909 evenly distributed SNPs which provided a high-density map of genetic markers with a mean density of approximately 1 per 56 bp (Supplementary Figure S4). Moreover, there were many low consensus regions due to the poor conservation of prophage genes. Strain RO-NN-1 lacked several mobile genetic elements that were present in strain TLPHMC/pBE2, such as ICE*Bs1* and prophage SPβ. The significant differences between RO-NN-1 and TLPHMC/pBE2 suggest that precise mapping of HGT events occurring between these two strains should be possible using next-generation sequencing.

**Figure 5.**
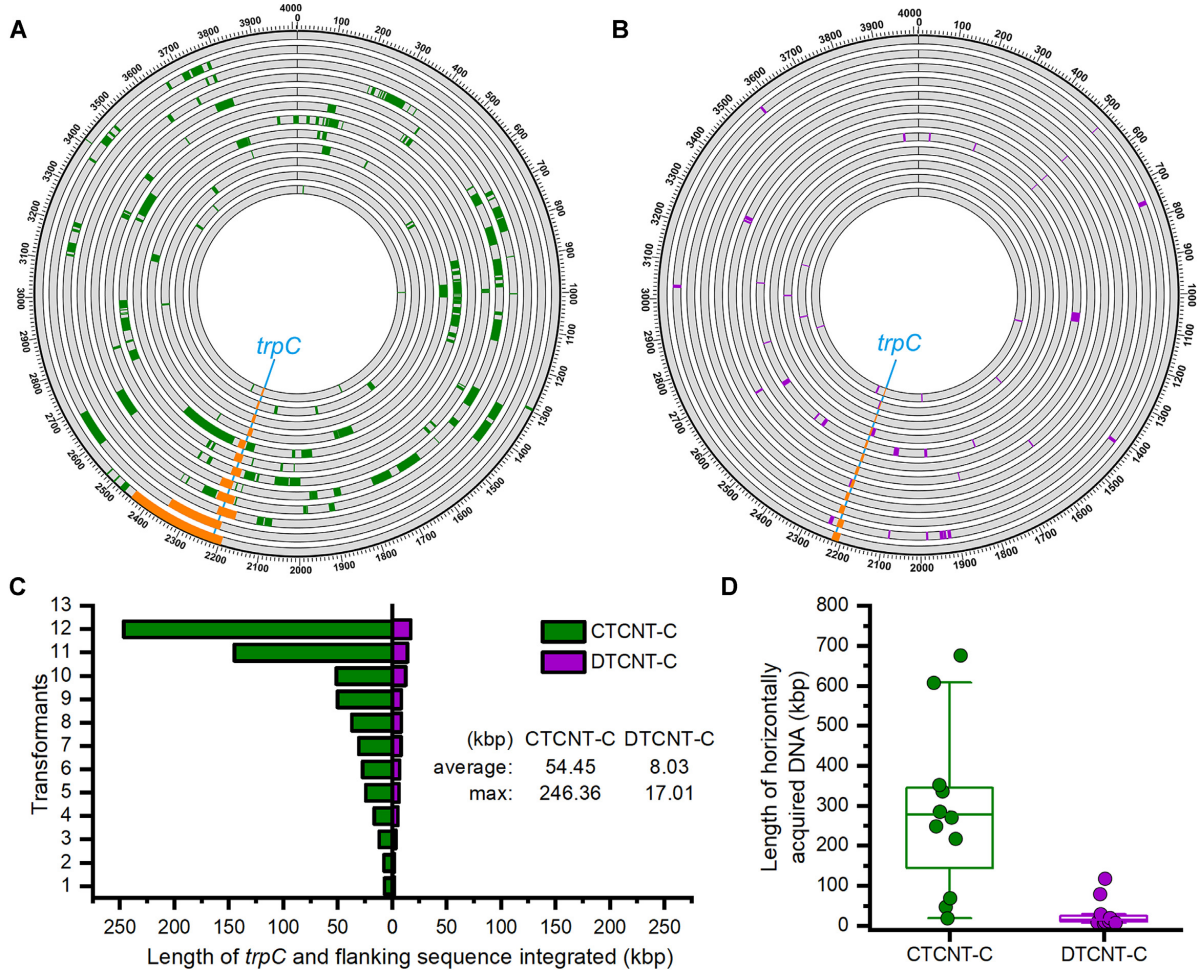
Quantification of transferred DNA segments in transformants based on whole-genome sequencing (WGS). (**A**) Circos plot of the acquired DNA sequence for each transformant generated by CTCNT-C (12 clones). Sequencing reads for each transformant were mapped onto the donor (RO-NN-1) genomes to visualize the transferred DNA regions. The outside black ring represents the donor chromosome labelled with 100-kb intervals; subsequent inner circles show the chromosome of transformants with the acquired DNA regions colored. The cyan line indicates the position of the amino acid marker (*trpC*) and the yellow arcs show the transferred region flanking *trpC*. Green arcs show the unselected transferred regions in the recombinants. (**B**) Circos plot of acquired DNA sequences from transformants generated by DTCNT-C (12 clones) mapped onto the donor (RO-NN-1) genomes as described in (A). Yellow arcs show the transferred region flanking *trpC*, whereas purple arcs show the unintentional acquired DNA regions (purple). (**C**) Comparison of the length of acquired DNA stretches containing the selected marker (*trpC*) of recombinant strains generated by CTCNT-C and DTCNT-C. The length of *trpC* containing DNA stretches was indicated on the X-axis for each transformant (Y-axis). The green- and purple bars represent transformants obtained by CTCNT-C and DTCNT-C, respectively. (**D**) Total length of transferred DNA segments of transformants generated by CTCNT-C and DTCNT-C. Green- and purple dots represent transformants obtained by CTCNT-C and DTCNT-C, respectively. The Y-axis indicates the total length of horizontally acquired DNA.

Strain RO-NN-1 was mixed with TLPHMC/pBE2 and transformants were selected on MM plates supplemented with kanamycin in the absence of tryptophan. Twelve *trp^+^* transformants from three independent experiments were chosen for WGS at a depth of ∼300× coverage. The generated FASTQ files of each transformant were compared with the reference genomes and mapped onto the genome of RO-NN-1 to visualize the transferred regions. For comparison, 12 transformants obtained by DTCNT-C (DTCNT using purified chromosomal DNA) using purified chromosomal DNA of RO-NN-1 were sequenced and analyzed in the same manner. As expected, all the transformants acquired the selected *trpC* marker from the donor strain RO-NN-1 (Figure 5A and B), regardless of whether they were obtained by CTCNT-C or DTCNT-C. The continuous DNA stretches flanking *trpC* in the transformants obtained by CTCNT-C ranged from 6.9 to 246.4 kb with an average length of 54.5 kb (Figure 5C). Notably, 67% of these acquired consecutive DNA regions were above 17 kb in the transformants obtained by CTCNT-C. By contrast, the continuous DNA stretches containing *trpC* from transformants obtained by DTCNT-C were much shorter, with the longest DNA segment reaching only 17 kb (Figure 5C). These results confirmed that CTCNT-C promotes the transfer of large continuous DNA segments, and correlated with our prediction that genetic markers within a distance of 62 kb can be co-transferred as continuous DNA stretches in CTCNT-C, whereas markers separated by a distance above 475 kb are likely co-inherited by independent transfer events (Figures 2C and 3B).

### Multiple transfer events are common in *B. subtilis* natural transformation

**Figure 6.**
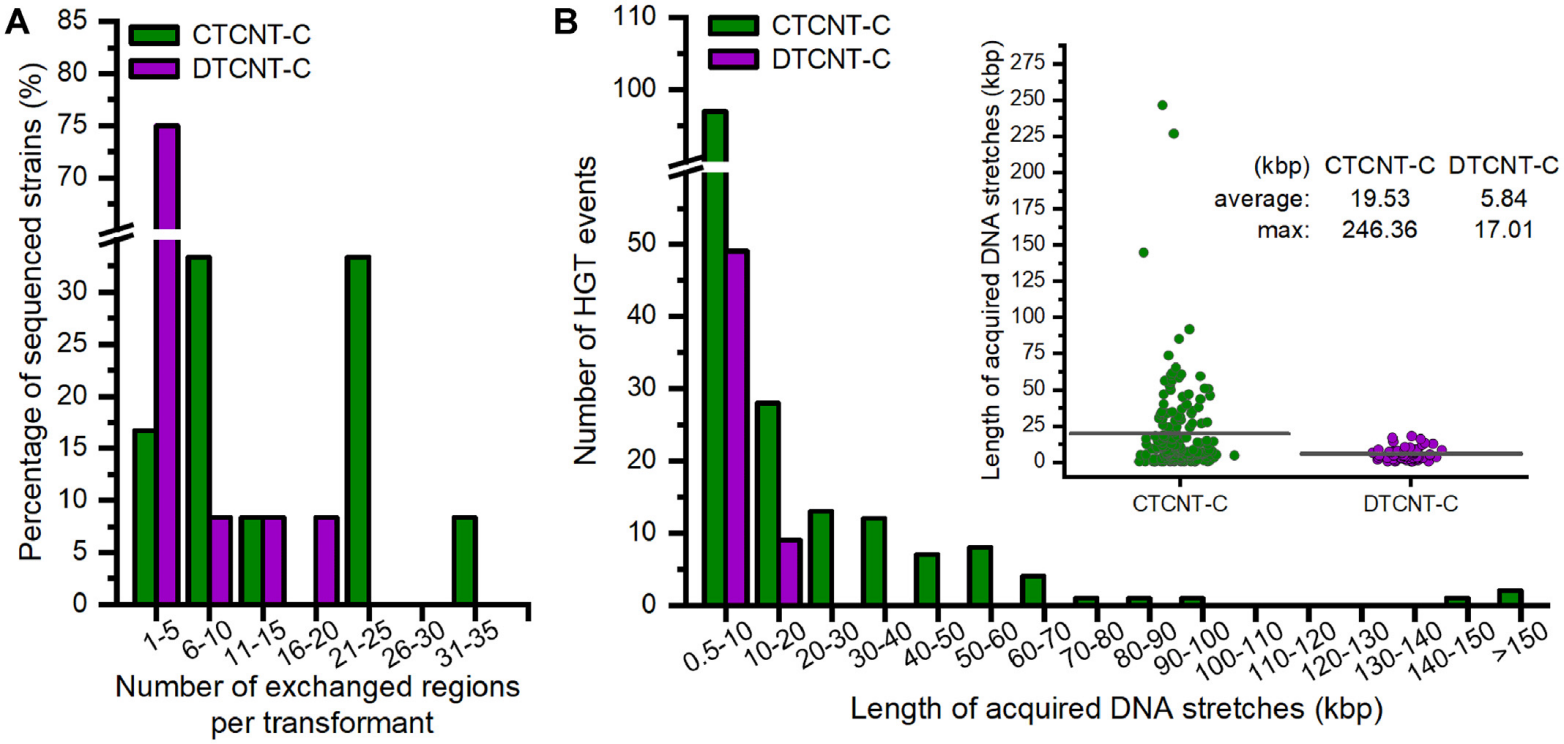
Quantification of horizontally acquired DNA in *B. subtilis* by WGS. (**A**) Multiple transferred DNA regions were identified in the transformant strains. The Y-axis shows the percentage of CTCNT-C transformants (green) and DTCNT-C transformants (purple) that exchanged one- or more DNA regions. (**B**) Long continuous DNA molecules were transferrable by CTCNT-C. The X-axis represents the length of individual consecutive DNA fragments. The green- and purple columns represent *B. subtilis* CTCNT-C and DTCNT-C, respectively. Inset: the length of per transferred segment; the transverse line corresponds to the average length.

Apart from the *trpC* locus, most of the transformants generated by CTCNT-C acquired substantial unselected segments from the donor strain (Figure 5A). The number, size, and location of these unselected integrated regions significantly varied among the transformants. All of the 12 transformants obtained by CTCNT-C experienced an additional 4–30 recombination events in addition to the selected *trpC* marker (Figure 6A). By contrast, the majority of transformants obtained by DTCNT-C experienced less than 5 recombination events, although a few underwent up to 17 (Figures 5B and 6A). The unselected integrated DNA segments obtained by CTCNT-C ranged from a few kb to > 200 kb with an average length of 16.96 kb, which was 31.1% of the length of the selected *trpC* region (Figure 6B). However, the unselected transferred regions in the transformants obtained by DTCNT-C ranged from only a few kb to 18.14 kb. Moreover, over 94% of them were below 15 kb. Nine of the transformants obtained by CTCNT-C acquired over 100 kb of DNA from the donor strain (average of 280 kb) if the selected *trpC* region was combined with the unselected transferred regions (Figure 5D). The maximum total size of acquired DNA reached 676.5 kb, or 16.86% of the donor genome in one transformant. By contrast, transformants obtained by DTCNT-C contained an average of 28.2 kb of donor genomic sequence, or approximately one tenth of the size obtained by CTCNT-C. Taken together, these data indicated that recombination events were very frequent at locations other than the selected marker in recombinant strains using *B. subtilis* CTCNT-C experiments. This also confirmed that double or multiple transformations were the dominant mechanism for the co-inheritance of genetic markers separated by a distance >500 kb as predicted above.

### CTCNT-C promotes large scale genome replacements in *B. subtilis* when multiple markers are selected

Donor-specific SNPs were distributed throughout the RO-NN-1 genome of the 12 sequenced transformants. The total combined length of the transferred regions reached 2.07 Mb, accounting for 51.58% of the RO-NN-1 genome when overlaps were calculated only once (Supplementary Figure S5A). This indicated that approximately half of the RO-NN-1 genome could be transformed. To determine the maximum number of HGT events and the maximum length of acquired genomic regions, multiple markers were selected simultaneously in *B. subtilis* CTCNT-C assay to generate recombinants for WGS: four transformants obtained with four selected markers and five transformants obtained with five selected markers were used for sequencing analysis.

**Figure 7.**
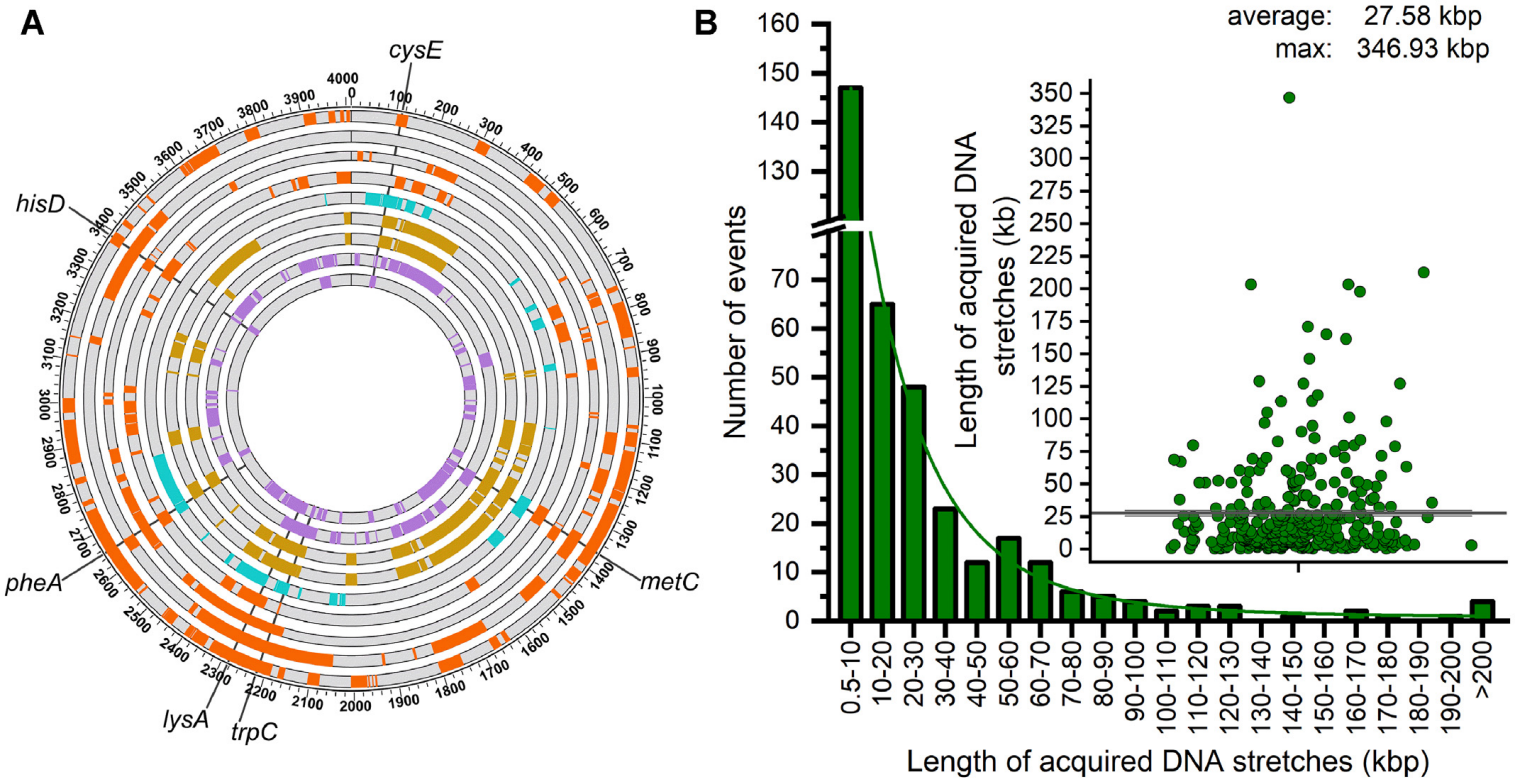
Quantification of acquired DNA from transformants obtained with multiple selected markers. (**A**) Graphic distribution of acquired DNA segments identified by WGS. Genome sequences of the donor were labeled in 100-kb intervals and marked with annotated genes. Each circle represents a sequenced transformant, and the acquired DNA were colored. Different colors represent different transformation groups. The purple circles were the transformants with the selected markers of *metC*, *trpC*, *lysA* and *hisD*. The yellow circles were the transformants with the selected markers of *metC*, *trpC*, *lysA* and *pheA*. The blue circle was the transformant with the selected markers of *cysA*, *metC*, *trpC*, *lysA* and *pheA*. The orange circles were the transformants with the selected markers of *metC*, *trpC*, *lysA, pheA* and *hisD*. (**B**) The number of transferred DNA segments and length of acquired consecutive DNA stretches of the transformants. The green line represents the best fit. Inset: the length of per segment; the transverse line corresponds to the average length of acquired DNA stretches.

Mapping of the transferred DNA segments onto the RO-NN-1 chromosome showed that the selected loci were exchanged as expected (Figure 7A). Recombination events at chromosome locations other than the selected marker were prevalent in transformants obtained with multiple markers. This was similar to the sequencing results of the transformants obtained using only one marker. The number of transferred regions significantly increased and the continuous transferred DNA segments were much larger in transformants obtained with multiple markers compared with those obtained using only one marker. Each transformant with multiple markers underwent over 22 recombination events with an average of 40 and a maximum of 66; this was five times more frequent than that obtained using one selection marker (Table 1). The average length of transferred continuous DNA segments reached 27.58 kb and the largest transferred segment was up to 346.93 kb (Figure 7B). Meanwhile, the total length of acquired donor DNA significantly increased, ranging from 0.7 to 1.5 Mb. In one transformant, 36.52% of the recipient genome (1,539.4 kb) was replaced by homologous sequences from the donor strain. The transferred regions covered >80% of the RO-NN-1 genome when the transferred DNA regions of the nine recombinants were combined and overlaps were only calculated once (Supplementary Figure S5B). In comparison, the average length of transferred DNA in transformants obtained with one marker was approximately 280 kb. Altogether, these results confirm that CTCNT-C promotes larger scale genome replacements compared with DTCNT-C, especially when multiple markers are selected simultaneously (increased metabolic stress).

**Table 1.** Summary of transferred donor-specific DNA segments in 9 recombinant strains obtained by CTCNT-C upon selection for multiple markers simultaneously

Clone^a^	Selected markers	Color of Figure 7A	HGT events	Max. recombination event size (kb)	Mean recombination event size (kb)	Total transferred DNA (kb)	Total transferred DNA, % of genome^b^
PC-1	*metC, trpC, lysA, hisD*	Purple	54	94.85	15.62	843.54	21.03
PC-2			54	165.10	24.23	1,308.24	32.61
HC-1	*metC, trpC, lysA, pheA*	Yellow	36	212.65	42.76	1,539.4	38.37
HC-2			35	203.28	38.60	1,351.06	33.68
H-1	*cysA, metC, trpC, lysA, pheA*	Blue	26	161.55	27.36	711.45	17.73
C-1	*metC, trpC, lysA, pheA, hisD*	Orange	66	146.23	22.67	1,495.93	37.29
C-2			31	170.85	24.82	769.37	19.18
C-3			22	346.93	49.29	1,084.31	27.03
C-4			32	82.73	22.35	715.13	17.82

^a^The letter(s) represent the unselected marker(s). P, *pheA*; C, *cysE*; H, *hisD*; two letters represent two unselected markers.

^b^Calculated percentage was based on the donor genome of RO-NN-1.

### Large-scale genetic linkage in the generation of transformants

Many of the transferred regions in each of the nine sequenced transformants obtained with multiple markers clustered in tracts (Figure 7A), occurring near long contiguous DNA stretches as well as in several short segments. A similar result was observed in the 12 sequenced transformants obtained with one selective marker. This indicated that clustering of the transferred regions was a common feature in recombinant strains obtained by CTCNT-C. These clustered regions may be derived from the same continuous DNA stretch transferred into the recipient cells. Alternatively, the long continuous DNA molecule could be cut into fragments after reaching the cytoplasm of the recipient cell, followed by multiple recombination events. In either case, the length of the transferred continuous DNA stretches should be larger than the calculated length from sequencing results. To estimate the size of the recombination tracts and the minimum number of independent recombination events in each transformant, we set up criteria for the recombination tracts: (i) the distance between adjacent acquired segments was <20 kb; (ii) acquired segments occupied at least 50% of the tract region and (iii) the maximum length of the tract region was <500 kb. These requirements were chosen because (i) the mean size of the transferred DNA of CTCNT-C was 19.53 kb so that two transferred segments separated by >20 kb are likely transferred by discrete fragments; (ii) previous studies showed that it was more rigorous if the transferred segments occupied at least 50% of the tract; (iii) co-transformation of two markers by a single cross-over was unlikely when the markers were separated over 500 kb as shown by genetic test and WGS.

**Figure 8.**
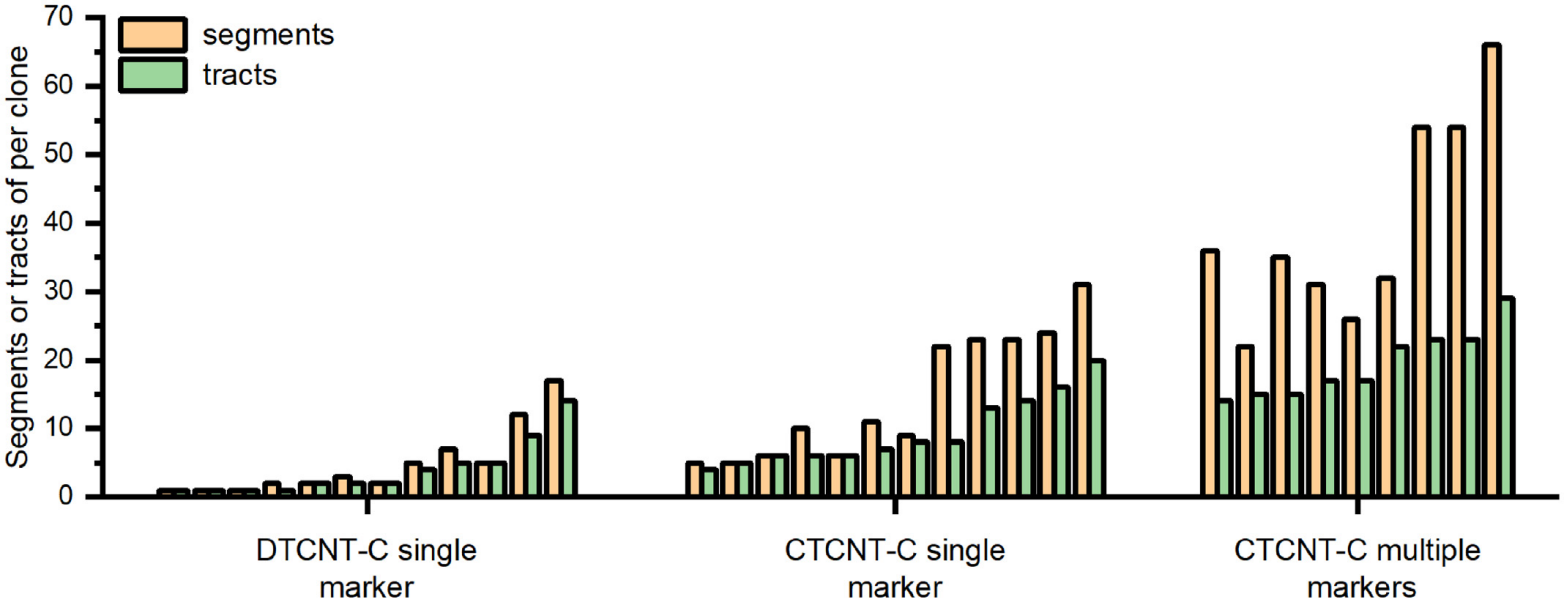
Comparison of the number of segments and recombination tracts (segments clustered to tracts) of recombinants. Columns show the number of segments or tracts of recombinants recovered from the three selective conditions indicated. Pale yellow columns and light green columns represent the acquired DNA segments of each transformant and the estimated tracts per clone, respectively. Each pair of columns describes an individually sequenced recombinant strain. The three groups of transformants were *trpC*^+^ recombinant strains obtained using purified genomic DNA (DTCNT-C single marker, 12 clones), donor cells (CTCNT-C single marker, 12 clones), and donor cells with four or five selective markers (CTCNT-C multiple markers, 9 clones).

Following consolidation of the transferred DNA stretches as recombination tracts, the estimated number of HGT events for each recombinant strain obtained by CTCNT-C with one and multiple markers decreased from 15 and 40 to 9 and 20 tracts, respectively (Figure 8 and Supplementary Table S3). By contrast, the estimated number of HGT events for recombinant strains obtained by DTCNT-C barely changed. Notably, the mean length of acquired continuous DNA segments in recombinant strains obtained by CTCNT-C with multiple selectable markers increased 1.3 times (63.65 kb/tract) due to the decreased number of recombination events. The total length of transferred DNA increased to 1,715.4 kb (42.76%) of the donor genome and the maximum size of a recombination tract was 418.5 kb. Based on these analyses, we proposed that the genetic linkage of transformation was defined by the length of contiguous DNA stretches and by the number of recombination events.

## DISCUSSION

Natural transformation is a major mechanism of HGT in bacteria which accelerates their evolution and enables adaptation to new environmental niches. In this study, we characterized cell-to-cell natural transformation in *B. subtilis* and tried to uncover the underlying mechanism. We found that co-inheritance of distantly localized genetic markers were common in recombinant strains obtained by CTCNT-C due to linkage of the markers in a large continuous DNA segment or transfer of discrete DNA fragments. In line with this, WGS of the recombinants revealed a frequent transfer of large genomic regions flanking the selective marker with an average length of approximately 54 kb (selection of one marker) in recombinant strains obtained by CTCNT-C. Moreover, we observed unbiased genome-wide transfer of unselected DNA regions in the recombinants of CTCNT-C. Strikingly, up to 37% of the donor genome was transferred into a single recipient when multiple markers were selected simultaneously in a single CTCNT-C experiment. These results indicate that the transfer of large genomic regions flanking the selected marker accompanied with the genome-wide transfer of unselected regions are responsible for the potency of CTCNT-C.

The transfer of large genomic regions rarely occurs during *B. subtilis* natural transformation with purified chromosomal DNA (DTCNT-C) extracted by a standard purification procedure. However, we previously found that CTCNT-C enabled high frequency co-inheritance of genetic markers separated by a distance of 130 kb (37), indicating that CTCNT-C facilitated the transfer of large continuous DNA regions. This study found that the ratio of co-inheritance of genetic markers within the range of 500 kb decreased significantly with increasing distance between the markers. However, if the distance was above 500 kb, the ratio of co-inheritance stayed constant at a lower frequency, which was still much higher than that of DTCNT. WGS revealed that the average length of continuous transferred DNA regions surrounding the selective marker in recombinant strains obtained by CTCNT-C was approximately 54 kb with a maximum length of 246 kb. By contrast, the length of transferred continuous DNA regions in transformants obtained by DTCNT-C were often below 10 kb, which is similar to previous reports (45,46). This suggests that genetic markers within a distance of 54 kb are frequently co-transferred into the recipient through a continuous DNA fragment, whereas genetic markers separated by a longer distance are more likely co-inherited by multiple transformations of discrete DNA fragments. This is supported by sequencing data of the recombinant strains obtained with multiple auxotrophic markers showing that most of the markers were transferred into the recipient as individual continuous DNA stretches with sizes ranging from a few kb to 347 kb. The ability to acquire large genomic regions through natural transformation using living cells as the source of transforming DNA was also observed in other naturally competent bacteria (19,26,27). For example, *V. cholerae* acquired up to 168 kb contiguous DNA segments when it was cultured on a chitin surface where interbacterial predation releases the DNA of the donor cells for the recipients (27). These results indicate that acquisition of large genomic regions is a common feature of naturally competent bacteria when living cells are used as the DNA donor.

Recombinant strains generated by CTCNT-C often experience additional multiple recombination events at locations of the chromosome that are separate from that of the selected marker. WGS of recombinant strains obtained with one selectable marker (*trpC*) showed that there were additional 4 to 30 HGT events in each recombinant. As a consequence, the average total length of acquired DNA in the recombinant strains was approximately 280 kb, with a maximum size of 676.5 kb, or 16.86% of the donor genome. Meanwhile, selection of four or five markers resulted in transformants undergoing an average of 40 recombination events and acquiring 0.7–1.5 Mb of DNA which accounted for 17–37% of the donor genome. Consolidation of the clustered recombination events into recombination tracts further increased the total length of acquired DNA in the recombinants. The acquisition of multiple unselected DNA regions is also reported in *V. cholerae* (27). These results indicate that the use of purified genomic DNA as the source of transforming DNA greatly underestimates the ability of natural transformation to promote HGT in *B. subtilis*, and possibly in other naturally competent bacteria. It is noteworthy that we also observed multiple recombination events in the recombinants obtained by DTCNT-C, but they were less common and only an average of 28 kb of DNA from the donor genome was transferred into a recombinant.

The effectiveness of CTCNT-C in *B. subtilis* HGT raises many interesting questions about natural transformation. For example, why does the use of living cells as the source of transforming DNA promote the exchange of genetic material, especially for the transfer of large genomic regions? One reason might be that the competent cells can acquire fresh DNA immediately after it is released from the donor cells which was previously proposed for *V. cholerae* and *S. pneumoniae* (23,25). Kelly and Pritchard showed that unlinked markers that are separated by a long distance could be delivered to the recipient cells simultaneously if the DNA was carefully extracted to avoid fragmentation (47). It is possible that in CTCNT-C assays large consecutive DNA molecules with high transforming ability are released by the donor cells. Moreover, these released DNA molecules may be in a protected form since CTCNT-C of *B. subtilis* is relatively resistant to DNase treatment (37). As a result, the presence of protected large continuous DNA molecules increases the local concentration of DNA on the surface of the recipient cells, enabling the transfer of large genomic regions containing the selected marker, and the transfer of many other genomic regions. It is noteworthy that the efficiency of plasmid transformation by CTCNT (CTCNT-P) is also much higher than that of DTCNT using purified plasmid DNA (DTCNT-P), as shown in Supplementary Figure S6. This suggests that the size of the transforming DNA molecules is not the only reason for the great efficiency of CTCNT-C in gene transfer. The duration of the competent state of the recipients may be another factor contributing to the efficiency of CTCNT-C. The exit of competence state may be delayed by the metabolic stress, especially when multiple auxotrophic markers were selected, giving the recipient cells sufficient time to uptake large DNA molecules and the occurrence of multiple recombination events. Lastly, the donor cells may also play an important role in CTCNT. We had shown before viable donor cells were required before the mixture of donor and recipient cells were plated on the selective medium (37). Also, close-proximity between donor and recipient cells were also required for CTCNT-C (37), suggesting that the gene transfer process was a coordinated process between the donor and recipients. In a preliminary study, we have found that the disruption of a number of genes significantly reduced the transformation efficiency of CTCNT, implying that the donor cells may be regulated under the selection pressure to provide highly transformable DNA for the recipients. Overall, the results in this study and previous reports indicate that using living cells as the source of transforming DNA is better than using purified DNA for both chromosomal and plasmid DNA transfer, but the underlying mechanism awaits determination.

A recent study found that kin-discrimination mediated killing at the meeting points of swarms of distinct *B. subtilis* strains releases DNA from the donor strain which significantly promotes transformation efficiency (24). The efficiency of CTCNT-C between two different 168 derivatives (kin) (Figures 1 and 2), or between RO-NN-1 and a derivative of 168 (non-kin) was similar, suggesting that kin-discrimination probably does not play an important role in CTCNT. Interestingly, in the above study the transformation efficiency was strongly affected by a SigW-mediated stress response in the donor cells, indicating that the donor cells play an important role in *B. subtilis* natural transformation. As mentioned above, viable donor cells are required at the beginning of the CTCNT assay. It will be of interest to test if the SigW-mediated stress response would affect CTCNT-C. Future experiments should also test if kin-discrimination mediated killing increases the number of transfer events and the length of transferred DNA segments at the encounter points of swarms of different *B. subtilis* strains.

Another interesting question raised by this study is how the recipient cells uptake such long continuous DNA stretches. The largest identified continuous transferred DNA segment was 347 kb, which was previously considered impossible using purified genomic DNA unless the DNA was extracted extremely carefully. However, our sequencing results clearly showed that the transfer of long continuous DNA segments was frequent in *B. subtilis* using CTCNT-C. If the DNA uptake apparatus transports DNA into the cytoplasm at approximately 80 bp/s as previously estimated (48), the transport of a continuous 347 kb DNA segment requires over one hour. Although the duration of the competence state is known to be altered by the environment (35,49,50) and the exit of competence state is likely delayed by the metabolic stresses in the CTCNT assay, it is hard to imagine that the uptake process takes more than an hour. It would be interesting to determine the rate of DNA uptake in the CTCNT-C experiments of *B. subtilis*, as well as in transformation of *V. cholerae* and *A. baumannii*, which can also acquire continuous DNA stretches over 100 kb (26,27).

A recent study used a derivative of 168 and RO-NN-1 (similar strains in our CTCNT-C) to investigate genome-wide recombination events of *Bacillus* species by protoplast fusion and observed frequent and unbiased recombination events throughout the genome despite of the genetic variability (51). Interestingly, the ability of CTCNT-C to transfer large DNA molecules appears to be greater than that by protoplast fusion. The gene transfer process in protoplast fusion is different from natural transformation in *B. subtilis*, as the former incorporates dsDNA directly, wheras the latter is a highly regulated process involving the processing of dsDNA into ssDNA and then integration. One possible mechanism is that in CTCNT, the recipient cells are primed (in the competent state) to take up DNA and for recombination, whereas in the case of protoplast fusions, the cells are not at a state that facilitates homologous recombination. As a consequence, although much larger genomic DNA is transferred from one cell to another in protoplast fusion, the efficiency for recombination of large DNA fragments is still lower than that of CTCNT. Although it is not clear if the recombinants generated by CTCNT-C can stably maintain the acquired DNA, the observation that recombination can occur effectively between lineages of *B. subtilis* with a substantial genomic diversity indicate that CTCNT-C may be a good way for *B. subtilis* genome engineering.

*B. subtilis* is a broadly adapted bacterial species that is able to grow in many different environments including soil, plant roots and the gastrointestinal tracts of animals. Comparative genomic analyses of different *B. subtilis* strains isolated from diverse environments revealed a remarkable genetic heterogeneity of this species (52). Natural transformation, along with transduction and conjugation, is believed to be the driving force for the evolution of *B. subtilis*. Here, we showed that CTCNT-C facilitates genome-wide genetic exchanges despite of the genetic variability between the strains. These observations indicate that *B. subtilis* can efficiently acquire exogenous DNA from distinct but closely related strains (98.08% ANI) occupying the same niches. In recent studies on *B. subtilis* natural chromosomal transformation, the intraspecies RecA-dependent transformation frequency decreased log-linearly with the increased nucleotide sequence divergence up to 15%, and reached a plateau at 23% of the species boundary (53). Interspecies chromosomal transformation with lower efficiency and cannot be detected beyond 8% of sequence divergence (54). CTCNT may maximize the incorporation of novel alleles and genes that are beneficial for its survival and growth, contributing to its successful adaption to various environments.

In summary, our study found that CTCNT-C significantly increases the number of transfer events in *B. subtilis* compared with DTCNT-C, enables the transfer of large genomic regions, and fosters large scale genomic exchanges. These features of CTCNT-C suggest that natural transformation may play an important role in promoting the evolution and adaption of *B. subtilis*. CTCNT-C may also be very useful for strain construction in industry as *B. subtilis* is an important strain for fermentation and biomedical research. On the other hand, this study, along with previous studies in *V. cholerae* and *A. baumannii*, indicates that the power of natural transformation in HGT is greatly underestimated using purified genomic DNA as the source of donor DNA. Therefore, further studies should re-evaluate natural transformation for many other naturally competent bacteria using healthy living cells as the source of transforming DNA.

## DATA AVAILABILITY

The scripts used for genome comparison are available on FigShare at https://doi.org/10.6084/m9.figshare.22081127.v1.

## ACCESSION NUMBERS

Genome sequences and raw sequencing reads of transformants are available in NCBI’s Sequence Read Archive (SRA) within PRJNA846324, SRA accession numbers SRR19569079 to SRR19569111.

## Supplementary Material

gkad138_Supplemental_File
