# Supplementary material for: Cell-to-cell natural transformation in Bacillus subtilis facilitates large scale of genomic exchanges and the transfer of long continuous DNA regions
Source: Nucleic Acids Res. 2023 Mar 13;51(8):3820–35. doi: 10.1093/nar/gkad138 (PMC10164588; doi:10.1093/nar/gkad138)
Supplement: gkad138_Supplemental_File [file gkad138_supplemental_file.pdf]

Supplementary Information for

Cell-to-cell natural transformation in *Bacillus subtilis* facilitates large scale of genomic exchanges and the transfer of long continuous DNA regions

Liping Deng, Chao Wang, Xiaoming Zhang, Wenjie Yang, Hao Tang, Xinru Chen, Shishen Du* and Xiangdong Chen*

State Key Laboratory of Virology, College of Life Sciences, Wuhan University, Wuhan 430072, China

* To whom correspondence should be addressed. Tel: +86-13476029961; Email: ssdu@whu.edu.cn. Correspondence may also be addressed to Xiangdong Chen. Tel: +86-13971423771; Email: xdchen@whu.edu.cn.

Supplementary Tables

Table S1. Bacterial strains and plasmids used in this study

Strains and plasmids	Genotype / description[a]	Source / reference
B.subtilis		
BG2036 (donor)	Δ*apr-684* Δ*nprE522* Km^S	(1)
BG2036Δ*comK* (donor)	Δ*comK* Δ*apr-684* Δ*nprE522* Km^S	(2)
168	*trpC2*	CCTCC[b](3)
CAmC (donor)	Δ*comK* Trp^+ *cgeD*::P_{pen}-*mCherry* Km^S	This study
SAmC (donor)	Δ*comK* Trp^+ *spoIVA*::P_{pen}-*mCherry* Km^S	This study
YAmC (donor)	Δ*comK* Trp^+ *ypuA*::P_{pen}-*mCherry* Km^S	This study
RO-NN-1 (donor)	Wild type Km^S	(4)
BR151	*trpC2 lysA3 metA10*	(5)
BR151/pBE2 (recipient)	*trpC2 lysA3 metA10* Km^R	(2)
TLPHMC	*trpC2* Δ*lysA* Δ*pheA* Δ*hisD* Δ*metC* Δ*cysE*	This study
TLPHMC/pBE2 (recipient)	*trpC2* Δ*lysA* Δ*pheA* Δ*hisD* Δ*metC* Δ*cysE* Km^R	This study
TLPHMC-G/pBE2	*trpC2* Δ*lysA* Δ*pheA* Δ*hisD* Δ*metC* Δ*cysE* *amyE*::P_{pen}-*lacI*Δ*11-gfpmut2* Km^R	This study
TLPHMC-G/pGK12H	*trpC2* Δ*lysA* Δ*pheA* Δ*hisD* Δ*metC* Δ*cysE* *amyE*::P_{pen}-*lacI*Δ*11-gfpmut2* Cm^R Erm^R	This study
E.coli		
DH5α	*supE44* Δ*lacU169* (φ80*lacZ*Δ*M15*) *hsdR17 recA1 endA1 gyrA96 thi-1 relA1*	Lab stock
GM272	*dam dcm hsdS21 metB1 galK galT22 mtl-2 ton tsx supE44*	(6)
Plasmids		
pBE2	*E. coli-B. subtilis* shuttle vector, Amp^R Km^R	(7)
pGK12	*E. coli-B. subtilis* shuttle vector, temperature-sensitive Cm^R Em^R	(8)
pUC18	Amp^R	Fermentas
pGK12H	pGK12 derivative with multiple-cloning site from pUC18	This study
pNNB194	*E. coli-B. subtilis* shuttle vector, temperature-sensitive Amp^R Erm^R	(9)
pNNB19-Δ*comK*	pNNB194 derivative containing homologous arms for	This study

	comK knock-out	
pNNB19-Δ*lysA*	pNNB194 derivative containing homologous arms for *lysA* knock-out	This study
pNNB19-Δ*pheA*	pNNB194 derivative containing homologous arms for *pheA* knock-out	This study
pNNB19-Δ*hisD*	pNNB194 derivative containing homologous arms for *hisD* knock-out	This study
pNNB194-Δ*metC*	pNNB194 derivative containing homologous arms for *metC* knock-out	This study
pNNB19-Δ*cysE*	pNNB194 derivative containing homologous arms for *cysE* knock-out	This study
pNNB19-*trp*	pNNB194 derivative containing *trp* open reading frame for tryptophan complementary strain	This study
pNNB194-mc-c	pNNB194 derivative containing homologous arms and P_{pen}-*mCherry* for deletion-insertion in *cgeD*	This study
pNNB194-mc-s	pNNB194 derivative containing homologous arms and P_{pen}-*mCherry* for deletion-insertion in *spoIVA*	This study
pNNB194-mc-y	pNNB194 derivative containing homologous arms and P_{pen}-*mCherry* for deletion-insertion in *ypuA*	This study
pNNB194-gfp	pNNB194 derivative containing homologous arms and P_{pen}-*lacIΔ11-gfpmut2* for deletion-insertion in *amyE*	This study

[a]Km, kanamycin; Cm, chloramphenicol; Erm, erythromycin; Amp, ampicillin; S, sensitive; R, resistant.

[b]CCTCC, China Center for Type Culture Collection.

Table S2. Primers used in this study

Primer ID	Description	Sequence[a] (5'-3')
Primers used for homologous flanking region to the gene deletion		
comK-US	Cloning *comK* upstream	CC**AAGCTT**TCCCCCCAATGCCTTTTTT
comK-UA	flanking fragment	*GTCCGCTCTTCTTTCGGGTACAGATTCTAAAGGTGCGTCTGTTT*
comK-DS	Cloning *comK* downstream	*AAACAGACGCACCTTTAGAATCTGTACCCGAAAGAAGAGCGGAC*
comK-DA	flanking fragment	CG**GGATTC**ACCAATTTGGACACCGCTGC
lysA-US	Cloning *lysA* upstream	CG**GAATTC**GATCTTCATAACGGACAA
lysA-UA	flanking fragment	*AAGCGCAGGACGAATATTGTCGCAAATTTCCGGAATGTCAAAAC*
lysA-DS	Cloning *lysA* downstream	*GTTTTGACATTCCGGAAATTTGCGACAATATTCGTCCTGCGCTT*
lysA-DA	flanking fragment	CC**AAGCTT**TAAATGGCAGATCAAG
pheA-US	Cloning *pheA* upstream	CG**GAATTC**TTGCTTTTGTTCCTTTGG
pheA-UA	flanking fragment	*GGGCCTAGAGCTCAATTTTGAATAGAAGCTGCCATATCATTGGC*
pheA-DS	Cloning *pheA* downstream	*GCCAATGATATGGCAGCTTCTATTCAAAATTGAGCTCTAGGCCC*
pheA-DA	flanking fragment	CC**AAGCTT**GTAAGACTGGTATGCACC
hisD-US	Cloning *hisD* upstream	CG**GAATTC**GAAGATGTTAAAGCAAAC
hisD-UA	flanking fragment	*ATGTCTTTTATGCCCAGCTCGGCAGATGAAGGATATGCGGCCGT*
hisD-DS	Cloning *hisD* downstream	*ACGGCCGCATATCCTTCATCTGCCGAGCTGGGCATAAAAGACAT*
hisD-DA	flanking fragment	CC**AAGCTT**ATCGTGTTCCGCCTGTGA
metC-US	Cloning *metC* upstream	C**AAGCTT**GTTCGTTACGCTTGCTAAAG
metC-UA	flanking fragment	*CGGCATCGAGACGCTCTTCAAGCTGAACCGGGTAAGAGATCAGC*
metC-DS	Cloning *metC* downstream	*GCTGATCTCTTACCCGGTTCAGCTTGAAGAGCGTCTCGATGCCG*
metC-DA	flanking fragment	T**GAATTC**TGGCGCCTGTTGCAAACTGT

cysE-US	Cloning *cysE* upstream flanking fragment	C**AAGCTT**ACGGGGGGAAGCATGTGTTT
cysE-UA		*AACCGAGCACTTTAGCACCTGTCCCAATGACAACCCCCATGCCG*
cysE-DS	Cloning *cysE* downstream flanking fragment	*CGGCATGGGGGTTGTCATTGGGACAGGTGCTAAAGTGCTCGGTT*
cysE-DA		CG**GAATTC**AATGTATTATAAAGTGTGATT
cgeD-US	Cloning *cgeD* upstream flanking fragment	CG**GGATCC**GACTGATTTGGTTATTTTCC
cgeD-UA		GG**GGTACC**GATACTTGTGAATCACCGCA
cgeD-DS	Cloning *cgeD* downstream flanking fragment	C**GAGCTC**ATCGCCGTTCGTTACTTAATC
cgeD-DA		CG**GAATTC**TCCTATATTGGAATAAATAT
spoIVA-US	Cloning *spoIVA* upstream flanking fragment	CC**AAGCTT**TTGGCCGACAACCCTGTCTA
spoIVA-UA		GG**TCTAGA**ATTGGAGATATCGCCAGAAG
spoIVA-DS	Cloning *spoIVA* downstream flanking fragment	CG**GGATCC**AGCAGCCTCATGAAATGGGA
spoIVA-DA		CG**GAATTC**TTGGAAAAGGTCGATATTTT
ypuA-US	Cloning *ypuA* upstream flanking fragment	CC**AAGCTT**GCCACAACGGTAACCGTGAC
ypuA-UA		CG**TCTAGA**GATTTTTGCAATCAGGGCTG
ypuA-DS	Cloning *ypuA* downstream flanking fragment	CG**GGATCC**GAAAAGCGCCGAAAAATCGG
ypuA-DA		CG**GAATTC**TCAAAAAGAAGTGCCGGGTG
amyE-US	Cloning *amyE* upstream flanking fragment	CC**AAGCTT**ATGTTTGCAAAACGATTCAA
amyE-UA		GC**TCTAGA**ATCAGACCAGTTTTTAATTT
amyE-DS	Cloning *amyE* downstream flanking fragment	CG**GGATCC**TCGACATGGATGAGCGATGA
amyE-DA		CG**GAATTC**GAAAGAATGTGTTACACCTG
Primers used for Trp^+		
trpAF-S	Cloning *trp*	GC**TCTAG**ATGAATTTCCAATCAAACAT

trpAF-A	open reading frame fragment from *B. subtilis* BG2036	CG**GGATCC**TCATTTTAAACTGTACAAGC
Primers used for fluorescent protein gene amplification		
mChrry-c-S	Cloning the P_{pen}-*mCherry* inserted into *cgeD*	GG**GGTACC**AAGCTAATTCCGTGGAAACGAG
mChrry-c-A		C**GAGCTC**TTATTTGTACAGCTCATCCA
mChrry-s-S	Cloning the P_{pen}-*mCherry* inserted into *spoIVA*	CG**GGATCC**AAGCTAATTCCGTGGAAACG
mChrry-s-A		GC**TCTAGA**TTATTTGTACAGCTCATCCATG
mChrry-y-S	Cloning the P_{pen}-*mCherry* inserted into *ypuA*	CG**GGATCC**AAGCTAATTCCGTGGAAACG
mChrry-y-A		GC**TCTAGA**TTATTTGTACAGCTCATCCATG
gfpmut2-S	Cloning the P_{pen}-*lacIΔ11*-*gfpmut2*	GC**TCTAG**AGTGGAAACGAGGTCATCATT
gfpmut2-A		CG**GGATCC**TTATTTGTATAGTTCATCCA
Primers used for identification of pNNB194-borne		
pNNB194-seqS	Identification of plasmid pNNB194	CAACTGTTGGGAAGGGCGAT
pNNB194-seqA		GCCTAACTGTTCTTTTGCAG
Primers used for identification of mutants		
comK-seqS	Identification of *comK* mutation	GCGTGTTGCGGGACTTC
comK-seqA		GCCCGATGCTGTTAGATTAC
lysA-seqS	Identification of *lysA* mutation	ACATTGTTCTTACACGGCAC
lysA-seqA		AAAACTTCATCCAGAAAGTC
pheA-seqS	Identification of *pheA* mutation	AAGTCGGTTATTTAGGTCCA
pheA-seqA		ACGAATGCTGCCTACTTATC
hisD-seqS	Identification of *hisD* mutation	CTTTCTCTCAAGCGTTCCATC
hisD-		TTCTACCATTGTTTCCGCCAC

seqA		
metC-seqS	Identification of *metC* mutation	ACATCGAATTTAGGATTTCC
metC-seqA		TCAAATTCGCCGTGAACAAG
cysE-seqS	Identification of *cysE* mutation	CTGCTTTTCAAACAGAGTGG
cysE-seqA		GCGCGTTCCCGATATGAATG
trpAF-seqS	Identification of Trp$^+$	TCACAACTTTATCCAAGCCA
trpAF-seqA		TCTTTCTTTTGGGTTTATTTG
trpC-seqS	Identification of *trpC*	AGCCGTGACGGTTTAGATGAG
trpC-seqA		GCCAGCGATAAAGCAGCGTTT
cgeD-seqS	Identification of *cgeD* mutation	ACAGAGATAAGGATAAGGGAG
cgeD-seqA		AATTGTCACTATGTCTTTTCTT
spoIVA-seqS	Identification of *spoIVA* mutation	TATACGGTAAATGACAAACAG
spoIVA-seqA		GAATCGCGAGCCCTGCCTTATA
ypuA-seqS	Identification of *ypuA* mutation	AGGTCAGTCTCGCGGATGCC
ypuA-seqA		AATTATCGAAGCTGTGAAAGA
amyE-seqS	Identification of *amyE* mutation	TCGGAGATTGGGATGATAGC
amyE-seqA		CGGAATCTCGCAGAATCAAG
Primers for red fluorescence determination		
cgeD-redfS	Identification of *cgeD*::P_{pen}-*mCherry* mutation	GACTGATTTGGTTATTTTC
cgeD-redfA		TCCTATATTGGAATAAATAT
spoIVA-redfS	Identification of *spoIVA*::P_{pen}-*mCherry* mutation	CGCGGATGATCAATACGCC
spoIVA-redfA		AGGTTTGCCAACCTCTTTCA
ypuA-redfS	Identification of *ypuA*::P_{pen}-	GAGCTTGGAGACAAAATCGG

ypuA-redfA	*mCherry* mutation	ATCTGCCATTTAAAACGGGT
Primers used for pGK12H construction		
MCS-S	Cloning multiple-cloning site	G**CCGG**TAAAACGACGGCCAGT
MCS-A		G**CCGG**CAGGAAACAGCTATGAC
Primers used in qPCR assays		
rpoB-S	Part of the reference gene *rpoB*	TGTCCGCATTGATCGCAC
rpoB-A		TCAAGCGTATTTCGCAGGTA
GK12H-S	Part of the chloramphenicol resistant gene on pGK12H	ACTGGTTACAATAGCGACGGA
GK12H-A		GGAAACAATTTCCCCGAACCA

[a]Restriction sites highlighted in bold. Italics stands for the overlap region for splicing by overlapping extension PCR (SOE-PCR).

Table S3. Donor variation segments clustered into recombination tracts.

Marker(s)	Total segments	Average segments per clone	Total tracts	Average tracts per clone	Total clusters	Average clusters per strain	% of strain with cluster(s)	Clusters spanned markers
DTCNT-C single	58	5	47	4	8	0.67	50	4
CTCNT-C single	175	15	113	9	36	3	75	4
CTCNT-C multiple	356	40	175	19	72	8	100	28

Marker(s)	% of marker spanned tract	Max. tracts per clone	Average transferred DNA per clone (kbp)	Max. transferred DNA per clone (kbp)	Max. transferred DNA per clone, % of genome[a]	Average length of segments (kbp)	Average length of tracks (kbp)	Max. length of tracks (kbp)
DTCNT-C single	33.33	14	31.74	121.55	3.03	5.84	8.1	37.2
CTCNT-C single	33.33	20	327.51	747.56	18.63	19.53	34.78	260.35
CTCNT-C multiple	68.5	29	1206.5	1715.4	42.76	27.59	63.65	418.51

[a]Calculated percentage was based on the donor genome of RO-NN-1.

Table S4. The counts of the transformants of Figure S2.

Selective plate	ΔT	ΔT-L	ΔT-LP	ΔT-LPH	ΔT-LPHM
Transformants[a]	>4000	>4000	3312±324	1236±118	511±42
Selective plate	ΔT-LPHMC	ΔT-P	ΔT-M	ΔT-H	ΔT-C
Transformants[a]	0	>4000	>4000	>4000	>4000

[a]The transformants on ΔT, ΔT-L, ΔT-P, ΔT-M, ΔT-H and ΔT-C plates were almost close together to many unintermittent CFU (colony forming unit) lines, single colony was difficult to identify. Therefore, the counts of the transformants were estimated. Figure S2C shows accurate ratio of co-inheritance of an unselected marker with *trpC*, in which the *trp*$^+$ transformants were diluted 1000-fold with PBS buffer and then plated for selection of *trp*$^+$ or double recombinants.

Supplementary Figures

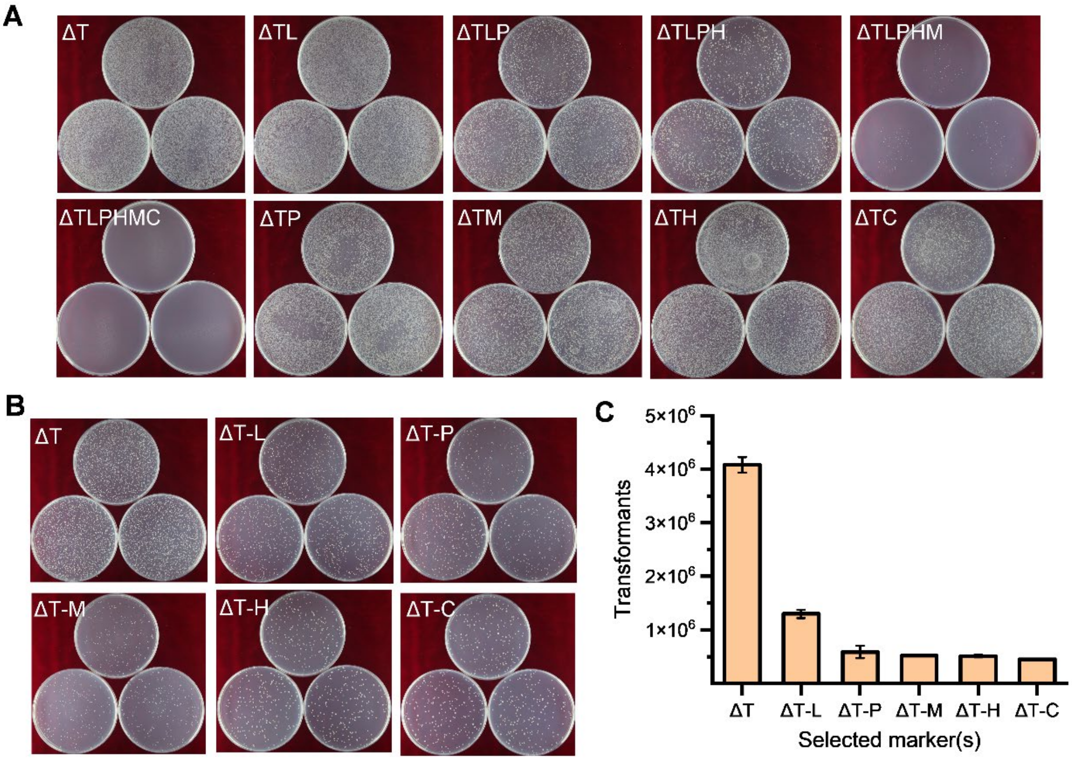

Figure S1. The co-inheritance of multiple genetic markers in *B. subtilis* CTCNT-C. The strain BG2036Δ*comK* was used as the donor, and TLPHMC/pBE2 was the recipient. The letter(s) on each plate represented the selected marker(s)： T for *trpC*, L for *lysA*, P for *pheA*, H for *hisD*, M for *metC*, C for *cysE*, multiple letters represent multiple selected markers. (**A**) Three parallel repetitions of CTCNT-C assays to test the co-inheritance of different kinds of selected markers, n=3. The recombinants were selected simultaneously by direct plating of the mixture of donor and recipient cells. (**B**) Co-inheritance of unselected marker with *trpC*. Transformants obtained on MM plates lacking Trp were pooled together, diluted 1,000 fold, and plated on selective plates lacking the indicated amino acids. (**C**)The number of *trpC* recombinants or double recombinants containing *trpC* and an additional marker. X-axis shows the selected marker(s), Y-axis shows the counts of colonies on the plates.

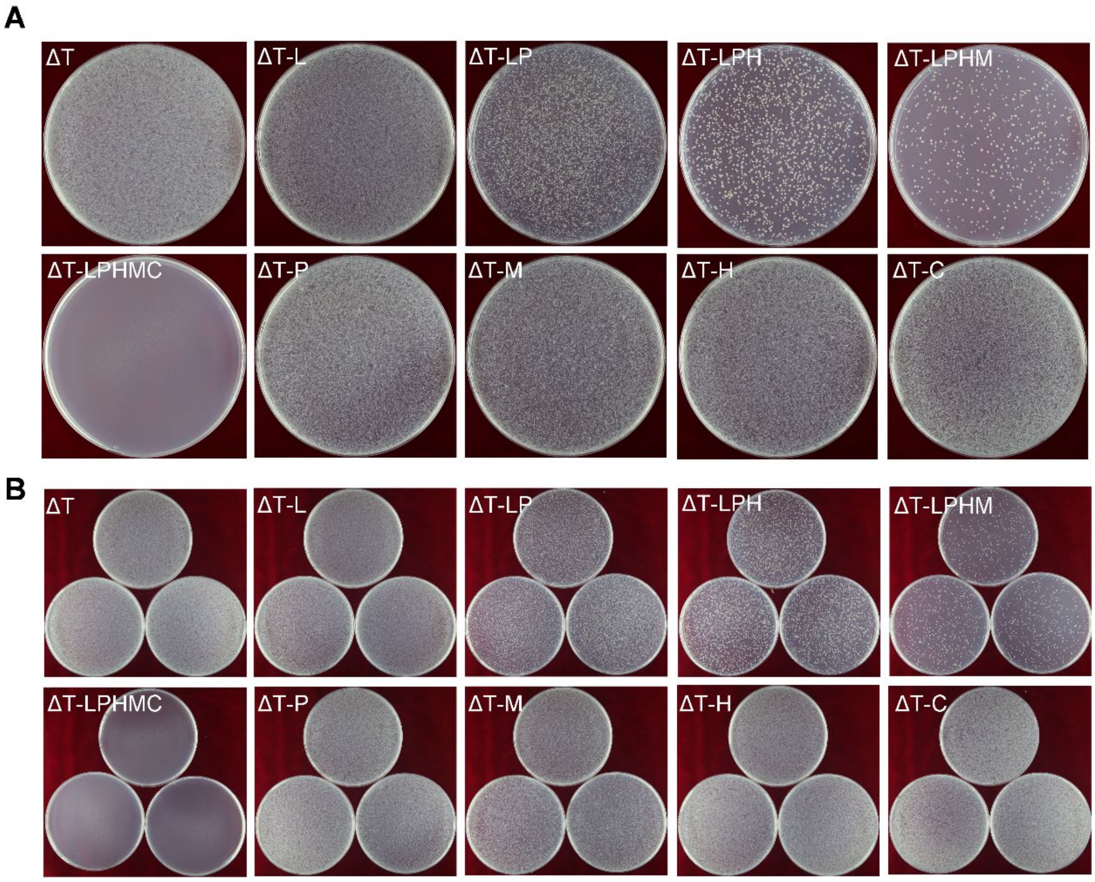

Figure S2. Co-inheritance of unselected markers with *trpC.* (**A**) Overnight suspensions of *trp*+ cells were plated on a series of MM plates with kanamycin in the absence of 1 to 6 amino acids required for the growth of the recipient strain. (**B**) Three repetitions of the co-inheritance of unselected markers with *trpC*. Parallel trials associated with panel A, n=3. The counts of the transformants were shown in Table S4.

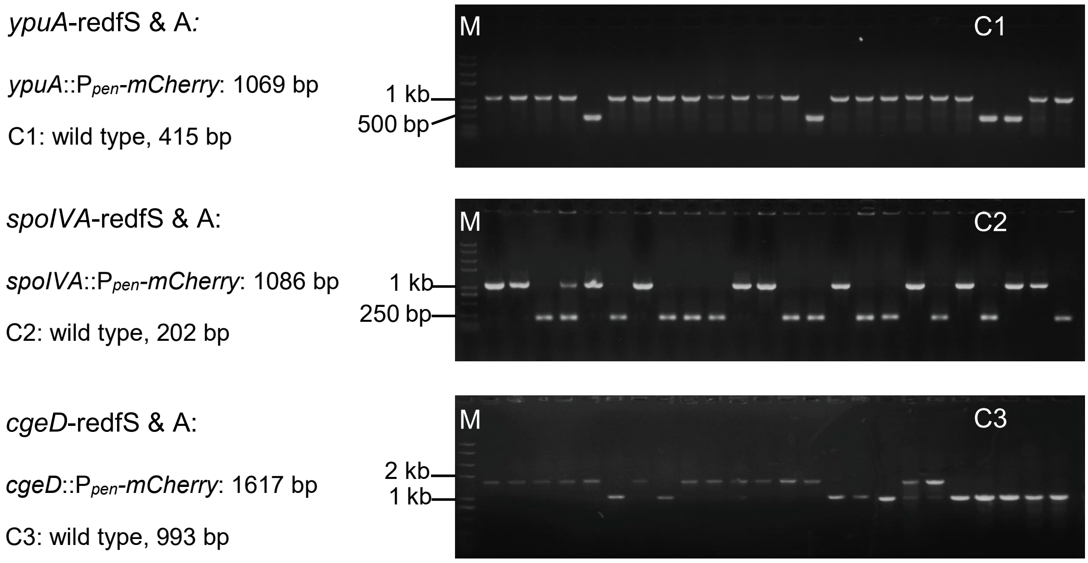

Figure S3. PCR analysis to check the co-inheritance of *mCherry* with selected markers. Colony PCR was carried out with primers complementary to the up and down region of the *mCherry* integrated loci and PCR products were analyzed by agarose gel electrophoresis. Approximately 100 transformants were tested in each set of experiments, and only partial results were shown. The first lane was loaded with the standard marker (M). The controls were amplified from *B. subtilis* 168.

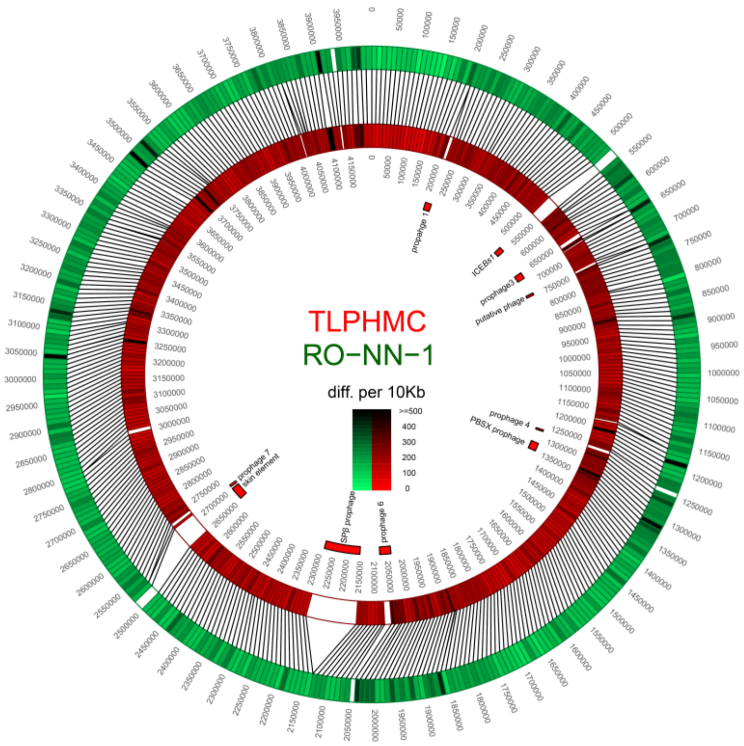

Figure S4. Comparative genomics of strain TLPHMC and the environmental isolate RO-NN-1. The genomic sequence of RO-NN-1 (green) was segmented in 10-kb fragments and aligned against the chromosome of the reference TLPHMC (red). To represent the differences between the two genomes, a color intensity scale was used that corresponded to the number of differences (SNP or indel), from 0 to >500 as measured per 10 kb fragment (same values are indicated for both colors). White regions show no homology. Several unique accessory genomic features of TLPHMC were highlighted inside the rings.

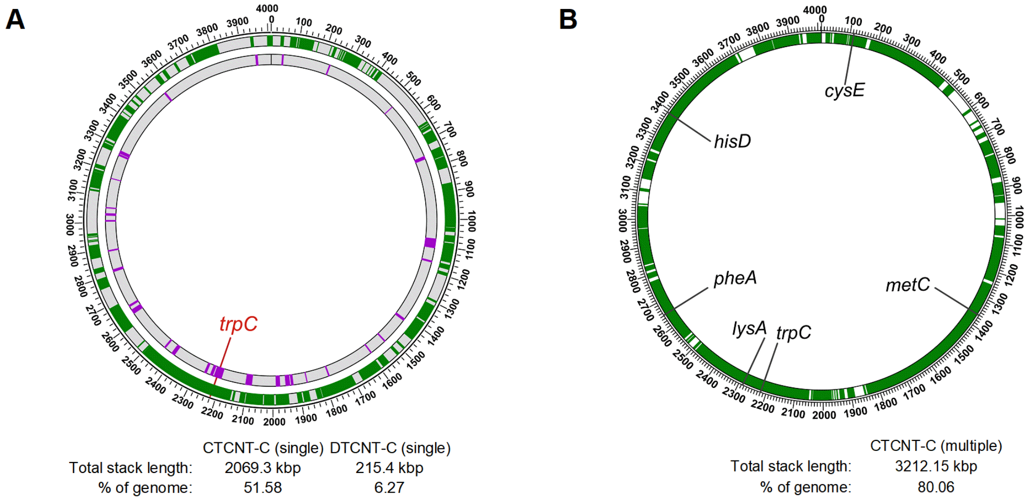

Figure S5. Distribution of the transferred DNA stretches of transformants in RO-NN-1 genome (overlaps only counted once). (**A**) Distribution of the transferred DNA regions in sequenced *trp*+ transformants obtained by CTCNT-C and DTCNT-C on the RO-NN-1 genome. The green blocks of the outer ring show the acquired DNA regions of the 12 sequenced transformants by CTCNT-C, the purple blocks of the inner ring represent the transferred DNA regions of 12 sequenced transformants obtained by transformation with purified DNA. The selected marker *trpC* was indicated by a red line. (**B**) Distribution of the transferred DNA stretches of all 9 sequenced transformants (selected with multiple markers) in RO-NN-1 genome. The green regions appeared in at least one transformant, labeled genes in the circle are the related markers.

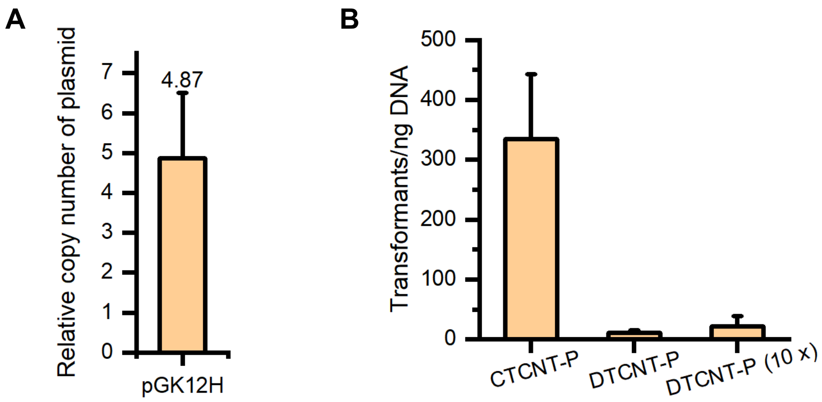

Figure S6. Live donor cells promote plasmid transfer in natural transformation. (A) Relative copy number of plasmid pGK12H in strain TLPHMC-G. (B) Comparison of the efficiency of natural plasmid transformation using live donor cells harboring the plasmid or purified plasmid DNA as the transforming material. The same concentration of plasmids within the cell culture or 10 times higher were used in DTCNT-P, recombinants were selected on MM with 50 μg/mL chloramphenicol and tryptophan.

Supplementary references

1. Yang, M.Y., Ferrari, E. and Henner, D.J. (1984) Cloning of the neutral protease gene of *Bacillus subtilis* and the use of the cloned gene to create an in vitro-derived deletion mutation. *J. Bacteriol.*, **160**, 15-21.
2. Zhang, X., Jin, T., Deng, L., Wang, C., Zhang, Y. and Chen, X. (2018) Stress-induced, highly efficient, donor cell-dependent cell-to-cell natural transformation in *Bacillus subtilis*. *J. Bacteriol.*, **200**.
3. Kunst, F., Ogasawara, N., Moszer, I., Albertini, A.M., Alloni, G., Azevedo, V., Bertero, M.G., Bessières, P., Bolotin, A., Borchert, S. *et al.* (1997) The complete genome sequence of the gram-positive bacterium *Bacillus subtilis*. *Nature*, **390**, 249-256.
4. Cohan, F.M., Roberts, M.S. and King, E.C. (1991) The potential for genetic exchange by transformation within a natural population of *Bacillus subtilis*. *Evolution; international journal of organic evolution*, **45**, 1393-1421.
5. Young, F.E., Smith, C. and Reilly, B.E. (1969) Chromosomal location of genes regulating resistance to bacteriophage in *Bacillus subtilis*. *J. Bacteriol.*, **98**, 1087-1097.
6. Palmer, B.R. and Marinus, M.G. (1994) The *dam* and *dcm* strains of *Escherichia coli*--a review. *Gene*, **143**, 1-12.
7. Guo XH, X.Z., Zhou M, Jia S, Xu Y. (1991) The construction of multifunctional shuttle vectors of *Bacillus subtilis*-*Escherichia coli*. *Chin J Biotechnol*, **7:224 –229**.
8. Kok, J., van der Vossen, J.M. and Venema, G. (1984) Construction of plasmid cloning vectors for lactic streptococci which also replicate in *Bacillus subtilis* and *Escherichia coli*. *Appl. Environ. Microbiol.*, **48**, 726-731.
9. Connelly, M.B., Young, G.M. and Sloma, A. (2004) Extracellular proteolytic activity plays a central role in swarming motility in *Bacillus subtilis*. *J. Bacteriol.*, **186**, 4159-4167.
